# Wide Band Gap Boron Nitride Nanodots‐Incorporated Polyetherimide Dielectrics for High‐Temperature Dielectric Energy Storage

**DOI:** 10.1002/advs.202520484

**Published:** 2026-01-12

**Authors:** Wen‐jin Hu, Wen‐hao Huang, Nan Zhang, De‐xiang Sun, Yong Wang, Jing‐hui Yang

**Affiliations:** ^1^ Key Laboratory of Advanced Technologies of Materials (Ministry of Education) School of Chemistry Southwest Jiaotong University Chengdu China

**Keywords:** electrostatic energy storage, high‐temperature, polyetherimide dielectrics, self‐healing, wide band gap

## Abstract

With the ongoing trend toward miniaturization in electronic devices and the concomitant increase in power density, the operational requirements for dielectric polymer films have extended beyond conventional room‐temperature conditions. In this study, low mass fraction boron nitride nanodots (BNNDs) were incorporated into polyetherimide (PEI) films. The strong π–π interaction between BNNDs and PEI enables BNNDs to effectively intercalate between adjacent PEI molecular chains, thereby effectively weakening the interchain conjugation effects within the PEI matrix, successfully suppressing energy losses at high temperatures. Consequently, the composite film achieved an exceptional breakdown strength (*E_b_*) of 549.4 MV m^−1^ at 200°C while maintaining discharge energy density (*U_d_*) of 6.49 J cm^−3^ and efficiency (η) of 65%. Furthermore, the film demonstrated notable self‐healing capability following dielectric breakdown, at 200°C and 500 MV m^−1^, the *U_d_* values before and after breakdown were 4.88 and 4.24 J cm^−3^, respectively, with η of 85% and 82%. In summary, this study demonstrates the existence of strong π‐π conjugated interactions between BNNDs and PEI molecular chains. Consequently, BNNDs can intercalate between PEI molecular chains, replacing the π–π conjugation between these chains, thereby enhancing the high‐temperature performance of aromatic dielectric polymers.

## Graphical abstract

The inherent conjugation effects between molecular chains in PEI dielectrics induce substantial energy loss escalation at elevated temperatures. This study incorporated BNNDs into PEI films, successfully achieving effective capture of motion charges under high‐temperature conditions. BNNDs effectively weaken the conjugated interactions between PEI molecular chains, thereby significantly enhancing the electrical insulation properties and energy storage capacity of PEI composite films.

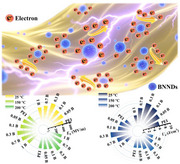

## 1 Introduction

Polymer dielectric films have found widespread application in advanced electronic devices owing to their superior charge–discharge rates compared to conventional energy storage systems [1, 2, 3, 4, 5]. Polymer dielectric films exhibit distinct advantages over ceramic materials, as they integrate excellent flexibility and processability with superior electrical insulation properties. Nevertheless, with the growing power requirements of modern electronics, polymer film capacitors are increasingly expected to operate under elevated temperature conditions. This operational scenario leads to substantial energy dissipation and a significant decline in dielectric insulation performance [6, 7]. However, conventional biaxially oriented polypropylene (BOPP) films, while widely used in commercial applications, exhibit insufficient thermal stability for high‐temperature operation [8]. As a result, high‐temperature‐resistant film capacitors have emerged as a prominent research focus in the field of dielectric materials. The molecular structure of polyetherimide (PEI), characterized by rigid aromatic moieties in its main chain, confers remarkable high‐temperature resistance [9, 10]. Despite its high glass transition temperature (*T*_g_), the presence of rigid aromatic rings in PEI induces pronounced conjugation effects within the molecular structure. This electronic delocalization leads to an exponential increase in energy losses at high temperatures, ultimately causing significant heat accumulation and reduced efficiency (η) [11, 12]. Consequently, there exists a critical need to engineer PEI dielectric films that simultaneously achieve high discharge energy density (*U_d_*) and minimal dielectric loss (tanδ).

Several strategies have been developed to enhance the high‐temperature energy storage performance of PEI films, primarily focusing on suppressing energy losses at elevated temperatures. One effective approach involves incorporating wide band gap inorganic fillers, such as BNNS [13], MgO [14], SiO_2_ [15, 16], titanium oxide clusters [17], etc., to limit the energy losses at high temperatures through the charge trapping effect of wide band gap nanoparticles. Moreover, in recent years, with the rapid advancement of metal‐organic framework clusters [18, 19], their application in polymer dielectric energy storage has also been steadily increasing [20, 21]. The main reason metal‐organic framework clusters can be applied is due to their high electron affinity, which effectively captures charges. However, when the mass fraction of filler in polymeric dielectrics is high, these inorganic nanofillers tend to form agglomerates, which can significantly compromise the mechanical flexibility and structural integrity of composites [22]. This aggregation phenomenon not only reduces the homogeneity of the polymer matrix but also creates localized stress concentrations that deteriorate the mechanical performance of the films. Additionally, constructing polymer dielectrics composed of alicyclic‐based with non‐conjugated structure has been widely explored recently, aiming to increase their intrinsic band gap (*E*_g_) of polymer dielectrics and reducing the electron delocalization [23, 24, 25]. The resulting polymers simultaneously achieve an effective promotion of both *T*_g_ and *E*_g_, showing great potential of polymer dielectrics applied in the high‐temperature conditions. However, a comprehensive study indicates that the thermally induced hopping conduction, especially the charge transfer associated with local chain transverse vibrations, is the predominant mechanism for energy losses at high temperature [26, 27]. Notably, recent studies show that such a charge transfer mechanism should be strongly dependent on the temperature rather band gap [28]. Thereby, only increasing the band gap by adding nanofillers/particles or molecular engineering is insufficient to address the energy losses at high temperature, and it is clearly difficult to further improve the energy storage under extremely high temperature (>150°C). Therefore, weakening the energy losses of dielectrics at high temperature has become a key issue for high‐energy storage and η. In particular, suppressing the interchain charge transfer under high temperature and electric field is still challenging.

In recent years, ultra‐fine nanodots, like carbon nanodots or semiconductor quantum dots, have attracted widespread attention in the field of dielectrics owing to their quantum effects and the resultant trapping of free charges behaviors [29, 30, 31]. Previous work focuses on the regulation of band gap via atom doping or chemical functionalization of nanodots [30, 31], in order to further enhance charge‐trapping capabilities under extreme operational conditions, thus increasing the energy storage capacity of polymer films. Nonetheless, boron nitride (BN) and its derived nanodots (BNNDs) constructed by strong B‐N covalent bonds are generally considered to have an extremely high band gap, as it is well acknowledged that BN exhibits a wide band gap of 4–6 eV [32, 33].

In this study, wide band gap boron nitride nanodots (BNNDs) were successfully prepared using a relatively green hydrothermal reaction method (Figure S1a). Subsequently, BNNDs were strategically introduced into PEI, significantly improving the electrostatic energy storage performance of PEI. Both experimental characterizations and theoretical models confirm that the strong π–π interaction between BNNDs and PEI enables BNNDs to intercalate effectively between PEI molecular chains. Consequently, the incorporation of BNNDs increases the interchain spacing in PEI composite films from 1.58 to 1.74 Å. Additionally, the wide band gap (5.51 eV) of BNNDs creates deep charge traps in the PEI film, which significantly suppresses charge transport under extreme operational conditions (Figure S2). Consequently, the breakdown strength (*E_b_*) of the composite membrane increased to 574.0 MV m^−1^ at 150°C, while energy loss was significantly reduced, achieving *U_d_* of 7.90 J cm^−3^ and η of 79%. Furthermore, when the temperature was raised to 200°C, the composite membrane exhibited a *E_b_* of 549.4 MV m^−1^, achieving *U_d_* of 6.49 J cm^−3^ and η of 65%. Finally, the PEI film exhibited intrinsic self‐healing properties, demonstrating remarkable electrical breakdown recovery with comparatively high *U_d_* reservation (4.24 J cm^−3^ post‐breakdown vs. 4.88 J cm^−3^ at 200°C and 500 MV m^−1^). From this study, it is of great novelty that BNNDs could be promising for suppressing the energy losses through intercalating into the PEI molecular chains, which offer significant potential for advanced energy storage applications under extreme operational conditions.

## 2 Results and Discussion

### 2.1 Theoretical Analysis of PEI Composite Films

**FIGURE 1:**
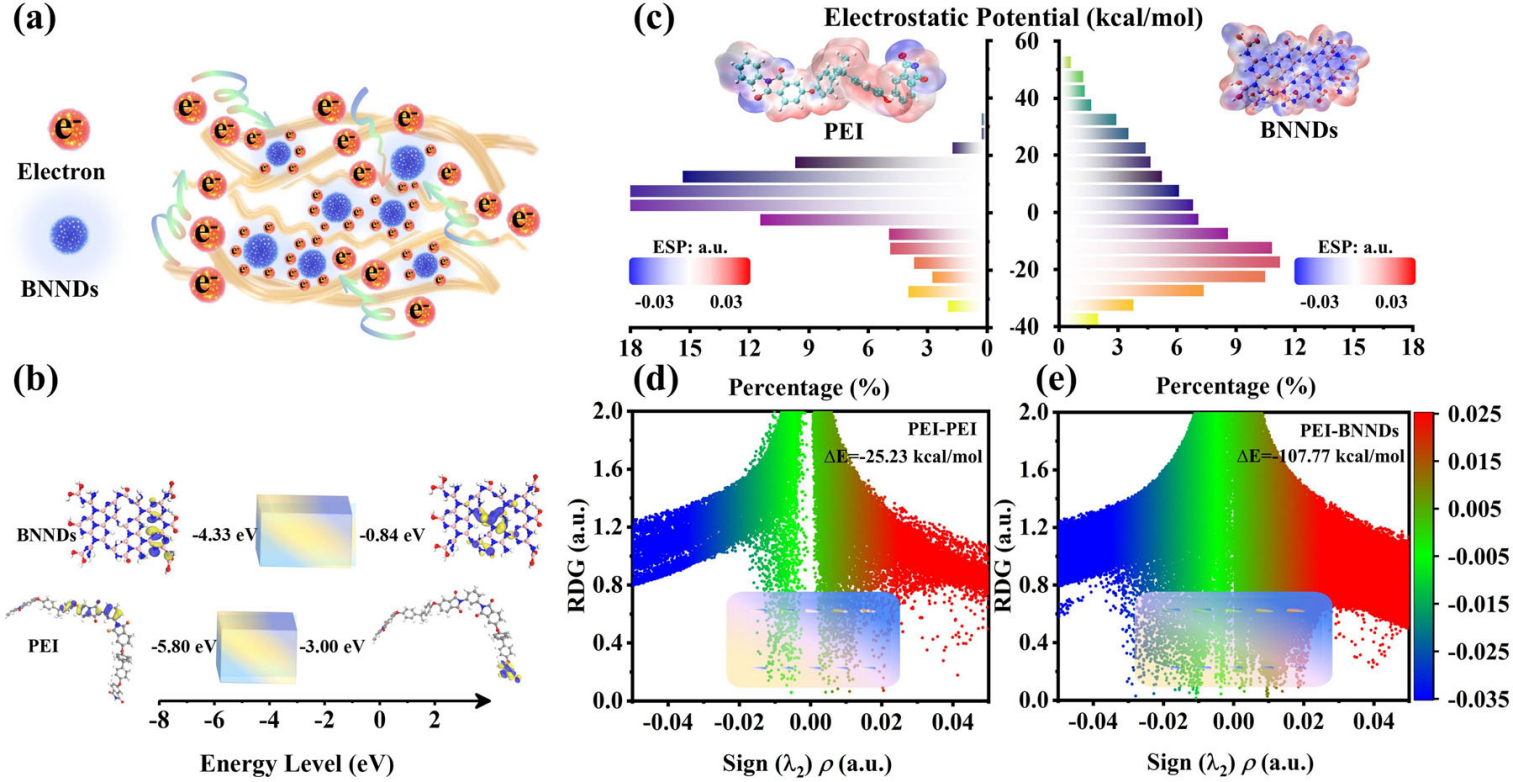
Mechanism Analysis of BNNDs Introduction into PEI. (a) The charge‐trapping effect of BNNDs in PEI composite films; (b) Band structures of PEI and BNNDs obtained via DFT calculations; (c) Electrostatic potential distributions of PEI and BNNDs; (d, e) Reduced density gradient (RDG) analysis of interaction energies.

It is well known that nanoparticles with a certain wide band gap can effectively trap charges under high temperatures and strong electric fields [14, 15, 16]. However, relying solely on wide band gap nanoparticles to enhance the high‐temperature resistance of dielectric films has its limitations. In this study, by incorporating wide band gap BNNDs between PEI molecular chains, the conjugated effects between these chains are weakened while their broader band gaps are utilized to trap charges generated from interchain motion (Figure 1a). To elucidate the mechanistic impact of BNNDs incorporation on charge transport in PEI, density functional theory (DFT) calculations were first performed to computationally characterize the electronic band structures of both BNNDs and PEI. As shown in Figure 1b, PEI has a band gap of 2.8 eV, whereas BNNDs possess a wide band gap of 3.49 eV, which facilitates efficient charge trapping from the PEI molecular chains. To further elucidate this charge confinement mechanism, the electrostatic potential (ESP) distributions of BNNDs and PEI were calculated, enabling quantitative analysis of local charge transfer trends and interfacial energy barriers. Figure 1c indicates that BNNDs possess a greater positive charge, enabling them to effectively attract and trap charges [34, 35]. Additionally, to analyze the structure‐activity relationship between BNNDs and PEI molecular chains, we calculated the interaction energies of PEI‐PEI and BNNDs‐PEI using DFT (Figure S3). As shown in Figure 1d,e, the interaction energy between PEI molecular chains is −25.23 kcal mol^−1^. However, due to the strong π–π interactions between BNNDs and PEI molecular chains, their interaction energy reaches −107.77 kcal mol^−1^ [36, 37], which is stronger than the π–π interaction energy between PEI molecular chains. At the same time, it also shows that BNNDs can effectively intercalate between PEI molecular chains, thereby weakening the conjugation effect between these chains and trapping charges.

### 2.2 Structural Characterizations of BNNDs and PEI Composite Films

**FIGURE 2:**
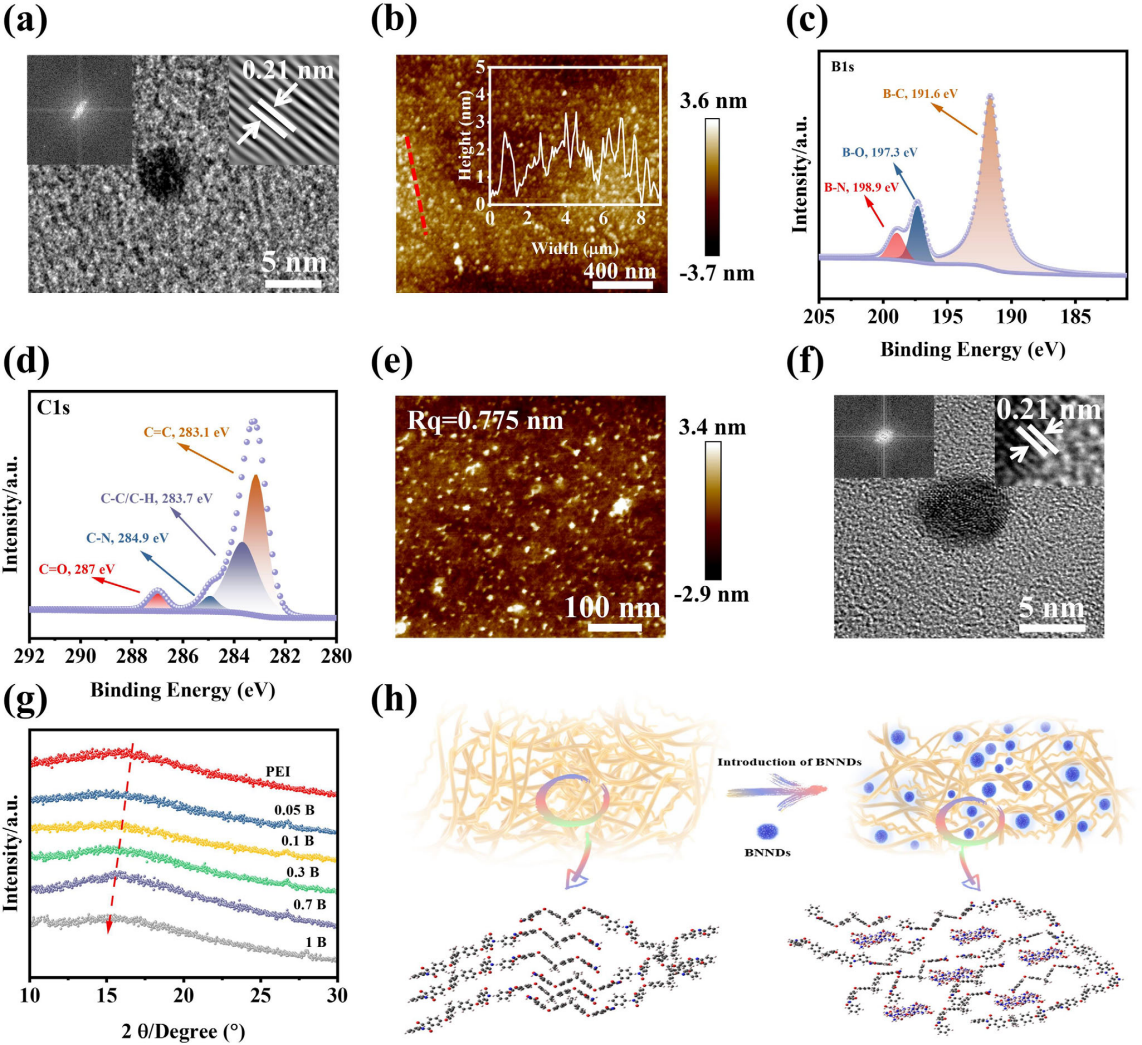
Morphological structure and internal mechanism analysis of PEI composite films and BNNNDs. (a) TEM image of BNNDs; (b) AFM image of BNNDs; (c) B1s high‐resolution fine spectra of BNNDs; (d) C1s high‐resolution fine spectra of 0.3 B film; (e) Surface AFM image of the 0.3 B film; (f) High‐resolution TEM image of BNNDs in PEI film; (g) XRD patters of PEI composite films; (h) Schematic diagram of the mechanism by which BNNNDs cause an increase in the intermolecular distance of PEI.

The structure and morphology of BNNDs were characterized in order to demonstrate whether BNNDs were successfully prepared. First, Figure 2a presents the high‐resolution TEM image of BNNDs, revealing their characteristic lattice spacing of 0.21 nm [38]. Additionally, the BNNDs exhibit a relatively uniform size of approximately 3 nm (Figure S4). Meanwhile, Figure 2b further shows that BNNDs are evenly dispersed. The crystalline structure of the synthesized BNNDs was further verified by X‐ray diffraction analysis (Figure S5a), exhibiting a well‐defined (002) diffraction peak at 25.3° [39], which indicates the successful preparation of BNNDs. The FTIR spectra in Figure S5b show that the stretching and bending vibration peaks of the B─N bond appear at 1349 and 775 cm^−1^, respectively [38, 40]. Simultaneously, C‐(B/N) and N‐B‐O peaks are observed at 1640 and 1096 cm^−1^, respectively. The XPS analysis of BNNDs is presented in Figure 2c and Figure S5c,d. The successful preparation of BNNDs is demonstrated by peak splitting of B1s, which reveals the presence of B─N, B─O, and B─C functional groups at 198.9, 197.3, and 191.6 eV, respectively. The above results indicate that all characteristic peaks of BN appear in the prepared BNNDs, indicating that BNNDs were successfully synthesized [38, 39, 41]. Furthermore, Figure S6 shows that the BNNDs have a wider band gap of 5.51 eV. This is mainly because of the presence of B and N atoms with different electronegativity in BNNDs, which endows them with good insulation capability [42]. They are able to act as charge trapping centers, trapping charges, and acting as charge scatterers.

The internal microstructure of PEI composite films has a significant effect on their dielectric properties, and thus, their structure and morphology features were analyzed. First, ATR‐FTIR tests were carried out on the PEI composite films containing different mass fractions of BNNDs (Figure S7a), and it is found that no new characteristic peaks appeared in the PEI composite films, which demonstrates that the introduction of BNNDs into the PEI films does not bring about any significant changes in the chemical structure of the PEI composite films [9]. In addition, the characteristic functional group peaks of PEI are found in Figure 2d and Figure S7b–d via XPS, with C‐N‐C peaks at 399 and 398 eV, respectively [17]. It is worth noting that no characteristic peaks of BNNDs are observed in the ATR‐FTIR and XPS tests of the PEI composite film. This further indicates that BNNDs have no effect on the molecular structure of the PEI matrix. The images in Figures S8 and S9 reveal that no obvious defects or pores can be observed in the interior of PEI composite films, which can effectively ensure structural integrity and defect‐freeness. Figure 2e and Figure S10 also show that the PEI composite films still maintain a low surface roughness, which is conducive to reducing the phenomenon of localized electric field concentration, allowing the composite films to maintain a good insulating ability as well as high breakdown strength [43]. Figure S11 provides the dispersion of BNNDs inside the 0.3 B film, and it can be found that the BNNDs are uniformly dispersed inside the film. Meanwhile, the characteristic crystalline diffraction fringes of BNNDs are also observed as 0.21 nm in the film (Figure 2f). Uniform dispersion of BNNDs in the composite film enables the film to inhibit electric field distortion induced by the aggregates of fillers. To further investigate whether BNNDs influence the structure of PEI, Figure 2g presents the XRD curves of the PEI composite films. It is observable that the amorphous diffraction peaks of PEI appeared in all XRD curves of PEI composite films [44], and it is evident that the diffraction peaks of PEI films exhibit a shift toward lower diffraction angles. Furthermore, the variations in molecular chain spacing within the PEI composite films were quantitatively determined using the Bragg equation [35, 45]. As illustrated in Figure S12, the molecular chain spacing of the PEI composite films first increases and then decreases subsequently when the mass fraction of BNNDs reaches up to 0.7 wt.%. Specifically, the molecular chain spacing expands from 1.58 Å in the pure PEI film to a maximum of 1.74 Å in the 0.3 B film. Furthermore, the molecular chain spacing of all PEI composite films exceeds that of the pure PEI film. This observation confirms that BNNDs can effectively enlarge the interchain distance of PEI via intercalating between PEI molecular chains, as shown in Figure 2h, which may in turn weaken the charge transport efficiency within the PEI matrix [44].

**FIGURE 3:**
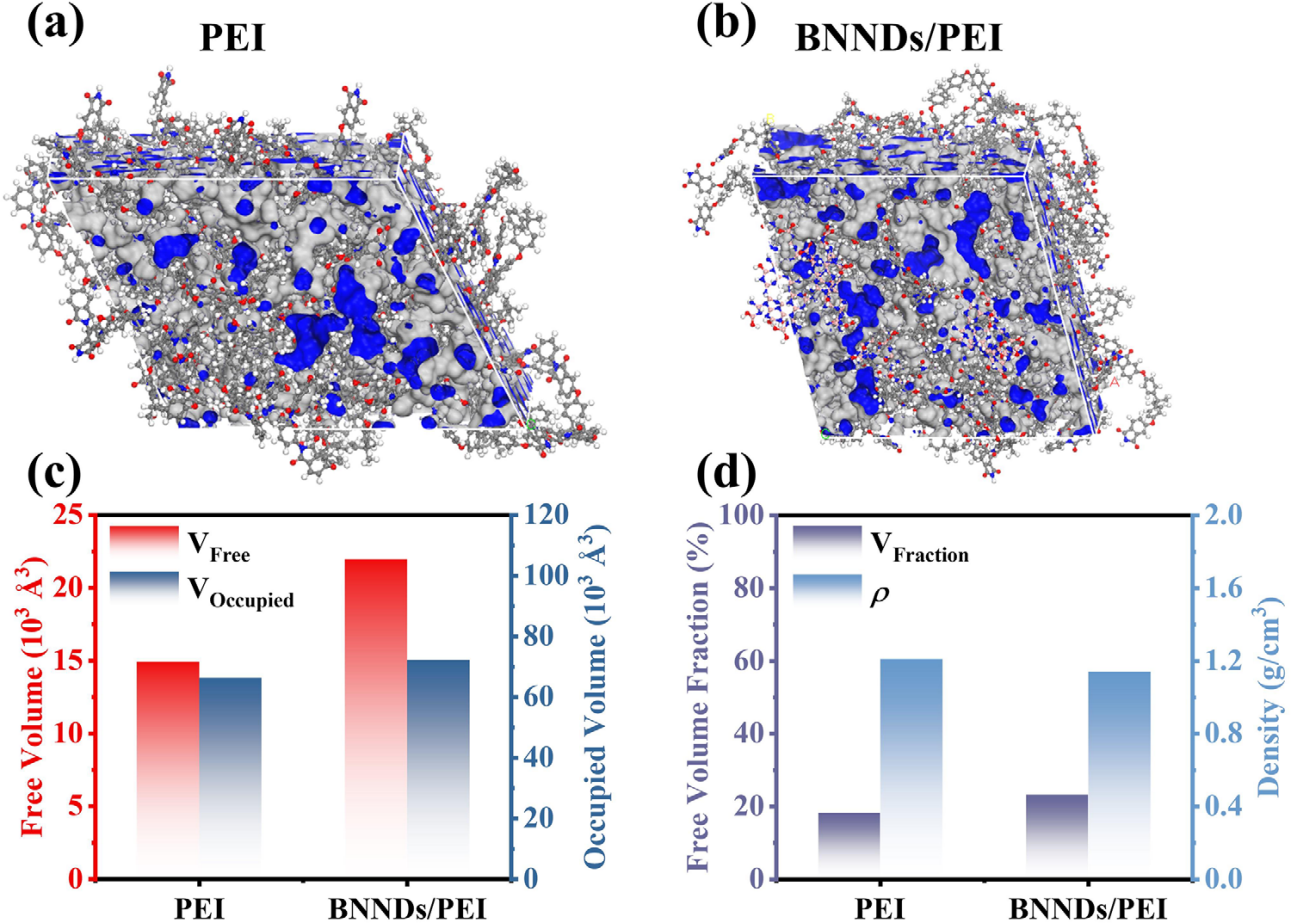
Molecular dynamics calculations of PEI composite films. (a) Model of PEI film; (b) Model of 0.3 B film; (c) Free volume and occupied volume analysis of composite film; (d) Free volume fraction and density analysis of composite film.

To further evaluate the free volume induced by BNNDs intercalated PEI molecular chains, the glass transition of PEI, as well as the thermal stability, was explored via DSC and TMA. As evidenced by Figure S13, the addition of BNNDs results in a slight decrease in *T*_g_ of the PEI composite films. In detail, the *T*_g_ of PEI is 209.6°C while the 0.3 B film decreases to 207.3°C. Besides, TMA characterization indicates that BNNDs incorporation in PEI films led to increased displacement (Figure S14), with the 0.3 B composite showing a CTE of 50.9 ppm °C^−1^. These thermomechanical response directly correlates with the BNNDs‐induced expansion of molecular chain spacing observed in XRD analysis. On the other hand, the values of *T*_g_ and CTE still remains at a relatively high level, and the slight variation ensures the thermal stability of composite films for high‐temperature applications. To further elucidate the effect of BNNDs doping on the PEI molecular chain, theoretical calculations were performed on the free volume of PEI and the composite film (Figure 3a,b). As shown in Figure 3c, the PEI film exhibits a relatively low free volume of 14 906.8 Å^3^ and occupied volume of 66 410.3 Å^3^, which can be attributed to its compact molecular chain spacing. In contrast, the PEI composite film containing BNNDs demonstrates significantly larger free volume of 21 964.9 Å^3^ and occupied volume of 72 249 Å^3^, resulting from its expanded molecular chain spacing. Figure 3d presents the free volume fraction and density characteristics of the PEI composite film. The results show a significant increase in free volume fraction from 18% for pure PEI to 23% for 0.3 B composite film, accompanied by a corresponding density reduction from 1.21 to 1.14 g cm^−3^, providing further evidence of expanded molecular chain spacing and a slight decrease in their *T*_g_. Such structural modification attenuates the conjugation effect between PEI molecular chains, consequently reducing energy losses at elevated temperatures.

### 2.3 Dielectric Properties and Electrical Insulation Behavior of PEI Composite Films

**FIGURE 4:**
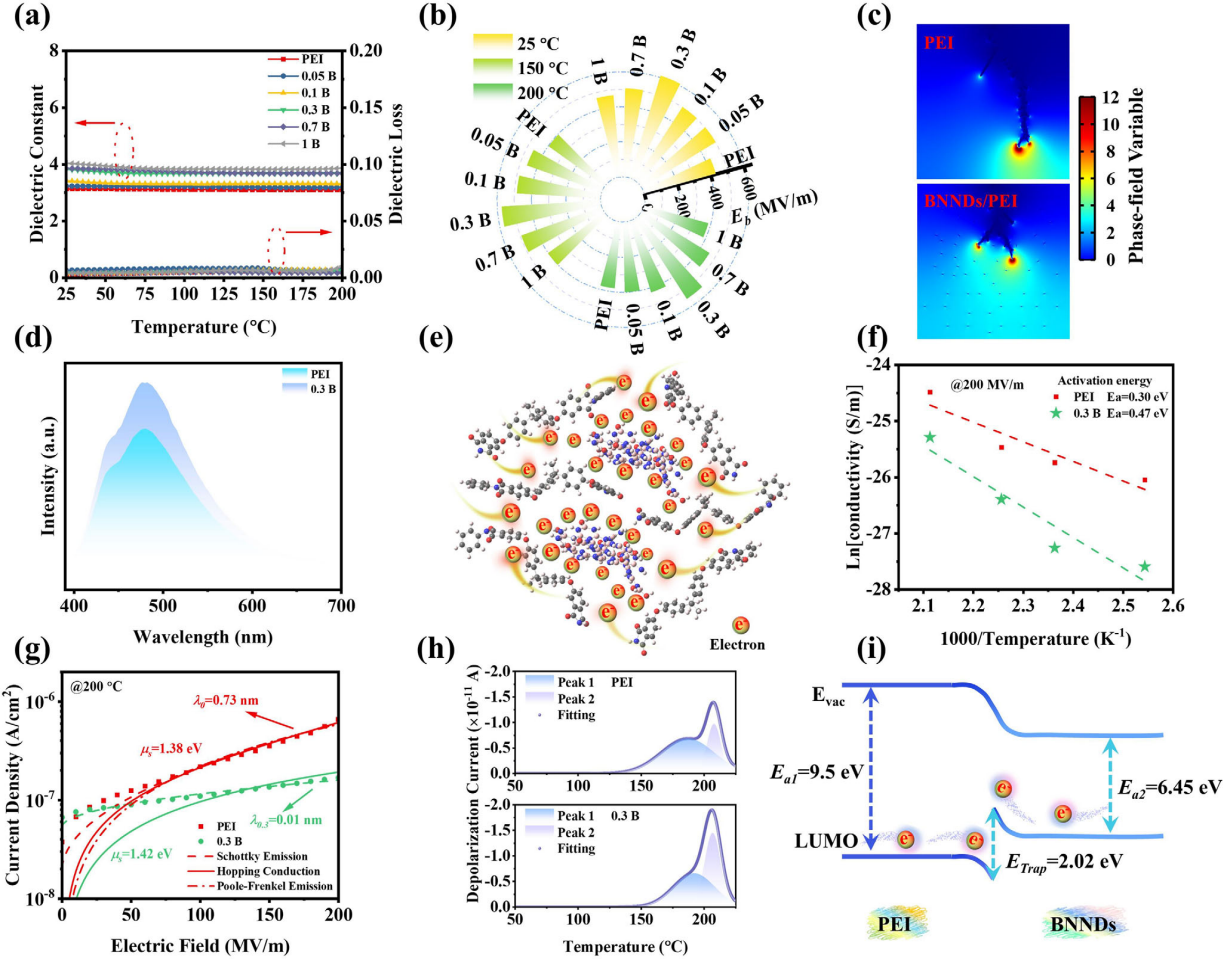
(a) Variable temperature dielectric spectrum of PEI composite films. Electrical insulation properties of PEI composite films. (b) *E_b_* analysis of PEI composite films at different temperatures; (c) Breakdown path of composite film at 0.5 s; (d) PL spectra of PEI and 0.3 B films with an excitation wavelength of 375 nm at room temperature; (e) The schematic showing of mechanism for BNNDs to inhibit charge transfer between PEI molecular chains; (f) Electronic activation energies of PEI and 0.3 B films; (g) Fitting of different theoretical models at high temperatures; (h) TSDC curves fittings of PEI and 0.3 B films; (i) Energy band diagram inside the composite film.

The dielectric properties of the films significantly influence their electrical insulation performance [46]. Notably, maintaining stable dielectric characteristics at elevated temperatures is critical for ensuring reliable insulation performance under high‐temperature operating conditions. Figure S15a shows that the dielectric constant of the composite films gradually increases with the increase in the content of BNNDs, and the dielectric constant increases from 3.1 for the PEI film to 4.3 for the 1 B film at 10^3^ Hz, which could be ascribed to the increased dipole motion capability owing to the increased intermolecular distance of PEI chains. On the other hand, BNNDs incorporation induces interfacial polarization at the BNNDs‐PEI interfaces [47]. Correspondingly, the tanδ shows a corresponding increase from 0.003 to 0.007 at 10^3^ Hz. However, the tanδ of all PEI composite films is below 0.01, which shows that PEI composite films are still able to maintain a low tanδ. Figure 4a presents the temperature‐dependent dielectric properties of the PEI composite films at 10^3^ Hz, covering a wide temperature range from 25°C to 200°C. It can be clearly found that all composites exhibit higher dielectric constants than PEI film while maintaining excellent stability as the temperature rises from 25°C to 200°C. Furthermore, all PEI composite films maintain low tanδ and conductivity (Figure S15c), ensuring their reliable performance across diverse application scenarios.

The high‐temperature insulation characteristics of the films critically determine their energy storage performance under elevated temperature conditions. The electrical breakdown behavior of the composite films at different temperatures is shown in Figure 4b and Figure S16. As the content of BNNDs increases, the *E_b_* of PEI composite films first increases and then decreases. It can be found that at 150°C, the *E_b_* of PEI film is 396.2 MV m^−1,^ and that of 0.3 B film is 574.0 MV m^−1^, which is an increase of 45% compared with PEI. Similarly, the *E_b_* of the 0.3 B film is 549.4 MV m^−1^ at 200°C, an increase of 57% compared with PEI. At high mass fractions, agglomeration of BNNDs within the PEI film creates breakdown weak points, resulting in reduced breakdown strength. However, it is observed that the breakdown strength of all composite films exceeds that of the PEI film because BNNDs effectively trap charge carriers migrating between PEI molecular chains. Since the film exhibits optimal electrical insulation properties at a mass fraction of 0.3 wt.%, subsequent characterization was conducted by comparing this sample with PEI films. Figure S17b indicates that the band gap of the PEI composite film increases to 3.38 eV after BNNDs incorporation compared to the PEI film. Additionally, BNNDs can be incorporated between PEI molecular chains. Under the combined effects of high temperature and strong electric fields, BNNDs effectively capture mobile charges from the PEI molecular chains (Figure S18). When the charge within BNNDs reaches saturation, the Coulomb blockade effect occurs. Consequently, BNNDs exhibit a repulsive effect toward additional charge while inducing charge scattering, thereby extending the migration path of carriers [48]. To further assess the enhancement of electrical insulation properties through BNNDs incorporation, phase‐field simulations were conducted on the composite system (Figure 4c; Figure S19). Figure 4c shows that, under the same conditions, the electrical dendrite growth rate of the PEI composite film is slower. In addition, the electrical dendrites in the PEI film penetrated through the entire film in 0.54 s, whereas the composite film took a longer time, 0.62 s (Figure S19). Phase‐field simulations indicate that introducing BNNDs as inorganic fillers into PEI effectively extended the path of electrical dendrites, thereby enhancing the electrical insulation properties of the composite film. The carrier transport behavior of polymer composite films at high temperatures is also one of the key factors determining their insulating properties. Elevated temperatures exacerbate charge carrier mobility and space charge accumulation, leading to increased energy losses and reduced breakdown strength. Effective modulation of these transport dynamics is essential for enhancing the high‐temperature reliability of polymer‐based dielectrics. In this study, intermolecular charge transfer occurs spatially between electron‐donating moieties (such as the aromatic rings of diamine units) and electron‐accepting moieties (such as the imide rings of dianhydride units) within the PEI molecular chains. During fluorescence emission, the energy of the incident photon is utilized to excite this process, forming an excited‐state charge‐transfer complex. The restricted movement of charge between molecular chains leads to an enhanced fluorescence effect in composite films, whereas charge movement within the molecular chains causes a red shift in the fluorescence of composite films [28, 49]. This study employed PL to further validate the charge‐transfer effect between PEI molecular chains. Suppressed charge transfer between molecular chains leads to enhanced fluorescence. Owing to their wide band gap (Figure 1b), BNNDs can capture charges from the PEI molecular chains, thereby enhancing the fluorescence emission intensity of the PEI composite films (Figure 4d). This enhancement indicates that BNNDs effectively suppress interchain charge transfer within the polymer matrix (Figure 4e), thereby significantly enhancing the insulation performance of the composite films. Moreover, as the temperature increases, it stimulates charge migration between molecular chains, resulting in reduced fluorescence intensity at elevated temperatures (Figure S20). Meanwhile, under the combined effects of electric field and temperature, the charge carrier transport behavior in the composite film exhibits activation energy dependence [13]. This electronic activation energy reflects the energy barrier height that charge carriers must overcome for conduction to occur. Figure 4f shows that the 0.3 B film has a larger electronic activation energy of 0.47 eV than the PEI film, confirming the superior charge trapping efficiency achieved through BNNDs incorporation [47]. To further characterize the high‐temperature deep‐trapping behavior, leakage current measurements were performed on the PEI composite films (Figure S21). It can be found that the leakage current is reduced for all the composite films. Figure 4g and Figure S22 present the high‐temperature leakage current characteristics of PEI and 0.3 B films. The observed charge transport behavior is analyzed using the following theoretical model [50]:

(1) $$J=2nq\lambda \nu *exp\left(-\frac{\mu_{H}}{k_{B}T}\right)*sinh\left(\frac{\lambda qE}{2k_{B}T}\right)$$

where *n* denotes the carrier density, *q* represents the carrier elementary charge, λ denotes the average hopping distance, *v* is the escape attempt frequency, µ_*H*_ is the activation energy associated with hopping conduction, *k_B_* is the Boltzmann constant, *T* denotes the absolute temperature, and *E* represents the electric field strength. The equation containing these parameters can be simplified as follows [51]:

(2) $$J=A*\sinh\left(B*E\right)$$

In the above equations, parameters *A* and *B* are defined as jump parameters, and they are essential for determining the jump dynamics of the carriers. At 150°C, Figure S22 shows that the PEI film has a jump distance of 1.03 nm, and the 0.3 B film has a lower jump distance of 0.29 nm. Similarly, at 200°C, Figure 4g shows that the 0.3 B film has a reduced hopping distance of 0.01 nm, which indicates a further increase in the trap depth of the film after the introduction of the BNNDs into the PEI film, resulting in the improved charge trapping ability of the composite film. Schottky analysis of the low‐field leakage current reveals that the 0.3 B film exhibits a significantly higher interfacial barrier height of 1.42 eV. This enhanced barrier and the shorter jump distance effectively suppress the injection of charges into the films (Figure S23). To quantify the charge trapping capability of the composite films, Figure 4h and Figure S24 present the TSDC curves of the PEI composite films. Notably, the 0.3 B film demonstrates a significantly enhanced depolarization current peak near 200°C. Furthermore, its charge trapping peak shifts to higher temperatures (192°C vs. 187°C for PEI), corresponding to an increased trap depth of 0.20 eV. These observations collectively confirm the deeper charge trapping capability achieved through BNNDs incorporation. In addition, the PEI film exhibits a trapped charge of 0.14 nC, whereas the 0.3 B composite demonstrates a significantly enhanced charge‐trapping capacity of 0.23 nC (Table S1). The increased charge capture capability of the composite film further indicates that BNNDs can capture charges between molecular chains. To further investigate the mechanism by which BNNDs influence charge transport, UPS measurements were performed on BNNDs and PEI (Figure S25). This revealed the valence band tops of BNNDs and PEI. Based on these results and the band gap of the PEI film, a band alignment diagram within the PEI composite film was constructed (Figure 4i). At the interface between PEI and BNNDs, a 2.02 eV interfacial potential barrier exists, which further inhibits charge transport at elevated temperatures. However, as the temperature‐induced electric field increases, thermally activated charges gradually accumulate, leading to enhanced charge mobility [7]. Ultimately, a greater number of charges overcome the interfacial potential barrier and are captured by the wide band gap BNNDs. The above phenomena indicate that wide band gap BNNDs can effectively suppress charge mobility, thereby enhancing the insulating properties of PEI composite films.

### 2.4 Capacitive Energy Storage Capacity and Self‐Healing Ability of PEI Composite Films

**FIGURE 5:**
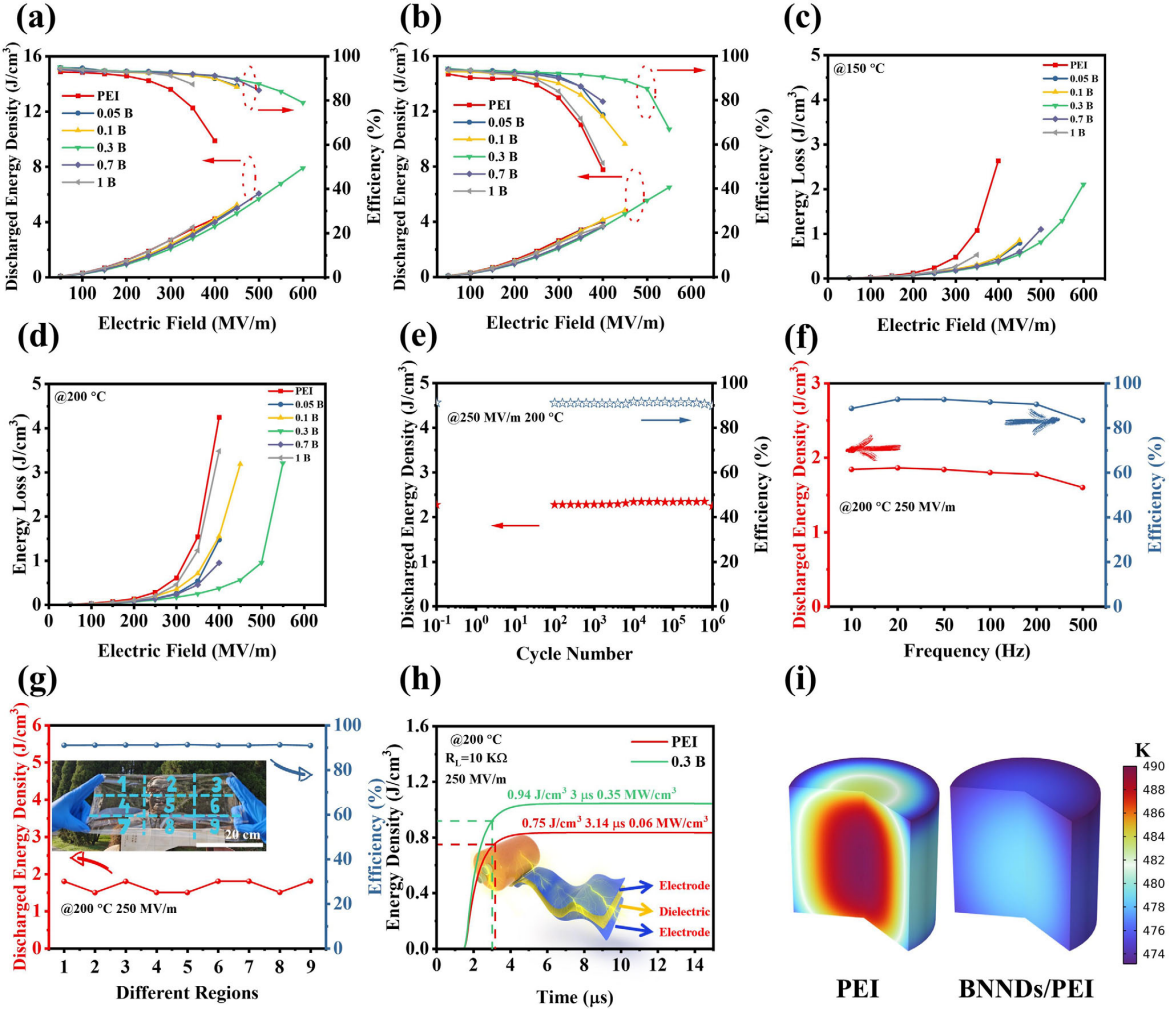
High temperature energy storage behavior of PEI composite films. (a) *U_d_* and η of PEI composite films at (a) 150°C and (b) 200°C; Energy losses analysis of PEI composite films at (c) 150°C and (d) 200°C; (e) Cycling stability of 0.3 B film at 200°C and 250 MV m^−1^; (f) Frequency dependence of energy storage of 0.3 B film at 200°C and 250 MV m^−1^; (g) High‐temperature energy storage behaviors under different regions of 0.3 B wide film; (h) Charging and discharging behaviors of PEI and 0.3 B film at 200°C and 250 MV m^−1^; (i) Simulation of capacitor steady state temperature distribution of PEI and 0.3 B films at 200°C.

The high‐temperature capacitive energy storage performance, a critical determinant of practical applications for film capacitors, was systematically evaluated through unipolar electric displacement–electric field (D–E) loops analysis across various temperatures (Figures S26–S31). At room temperature, Figure S30 shows that the energy density of the composite film first increases and then decreases with increasing BNNDs mass fraction. At a mass fraction of 0.3 wt.%, *U_d_* of 8.58 J cm^−3^ and η of 91% are achieved. This is primarily because BNNDs can effectively capture charges from the PEI molecular chains. However, BNNDs with high mass fraction aggregate within the film, resulting in significant energy loss and a gradual decrease in energy density. At the same time, the incorporation of BNNDs into PEI composite films significantly enhances their high‐temperature energy storage performance (Figure 5a,b). At 150°C, the PEI film exhibits *U_d_* of 4.25 J cm^−3^ and η of 62%, whereas the 0.3 B film demonstrates a high *U_d_* of 7.90 J cm^−3^ and η of 79%. At 200°C, the PEI film exhibits *U_d_* of 4.01 J cm^−3^ and η of 49%, while the 0.3 B film achieves a high *U_d_* of 6.49 J cm^−3^ and η of 65%. Additionally, the residual polarization of the film under the maximum electric field directly influences its energy storage behavior. It can be observed that at elevated temperatures, the 0.3 B film exhibits less residual polarization than the PEI film, indicating lower energy loss (Figure S29b,c). These results confirm the critical role of BNNDs in improving capacitive energy storage properties under high temperatures. It can be observed that the composite film exhibits reduced energy loss. For instance, at 400 MV m^−1^ and 150°C, the energy loss of the PEI film is 2.63 J cm^−3^, whereas that of the 0.3 B film is 0.36 J cm^−3^ (Figure 5c). Similarly, at 400 MV m^−1^ and 200°C, the energy loss of the PEI film is 4.25 J cm^−3^, whereas that of the 0.3 B film is 0.38 J cm^−3^ (Figure 5d). This is primarily attributed to the wide band gap of BNNDs and the diminished conjugation effect between PEI molecular chains.

Furthermore, the reliability and service life of polymer film capacitors are crucial for their application scenarios. Nevertheless, the operational reliability and lifetime of polymer capacitor films are critically dependent on their cyclic stability and frequency‐dependent performance characteristics [22]. Cycling performance tests of the 0.3 B film at elevated temperatures are given in Figure 5e and Figure S32, where it can be observed that the film still maintains a better energy storage behavior with increasing number of cycles, while maintaining an η close to 90%. The frequency‐dependent energy storage performance of the 0.3 B composite film is presented in Figure 5f and Figure S33. The results demonstrate exceptional frequency stability, with less than 5% variation in discharge energy density across the 10‐500 Hz range. This remarkable stability stems from the carrier confinement of the BNNDs and the weakened conjugation effect in the PEI. Next, in order to assess the mass stability performance of the film, different regions of the 0.3 B film were tested for energy storage behavior (Figure 5g; Figure S34). At 200°C, Figure 5g shows that the film exhibits an energy storage capacity of 1.5–1.8 J cm^−3^ at 250 MV m^−1^ while maintaining nearly η of 90%, demonstrating that the film is suitable for large‐size preparation while having good performance stability.

Subsequently, the discharge capability of the composite film as a capacitor film was further evaluated (shown in the inset of Figure 5h; Figure S35). Figure 5h shows that the 0.3 B film has an increased *U_d_*, with a time of 3 µs at *U_d_* above 90%, demonstrating the ultra‐fast discharge rate of the 0.3 B film, which is one of the advantages of polymer film capacitors [52]. Additionally, the 0.3 B film exhibits a pulse power density of 0.35 MW cm^−3^, which is higher than that of the PEI film, indicating the potential application of composite films in power pulsers. During operation, capacitors inevitably experience energy dissipation through energy losses, leading to significant heat generation. This thermal loading substantially degrades device performance and accelerates aging mechanisms, ultimately limiting operational lifetime [53]. Therefore, a temperature steady state simulation of the interior for a capacitor consisting of film was carried out (Figure 5i). The temperature analysis reveals that the 0.3 B film has a reduced internal temperature, which provides for stable operation at high temperatures and high electric fields, and this also shows the potential of the film for application as a capacitor.

**FIGURE 6:**
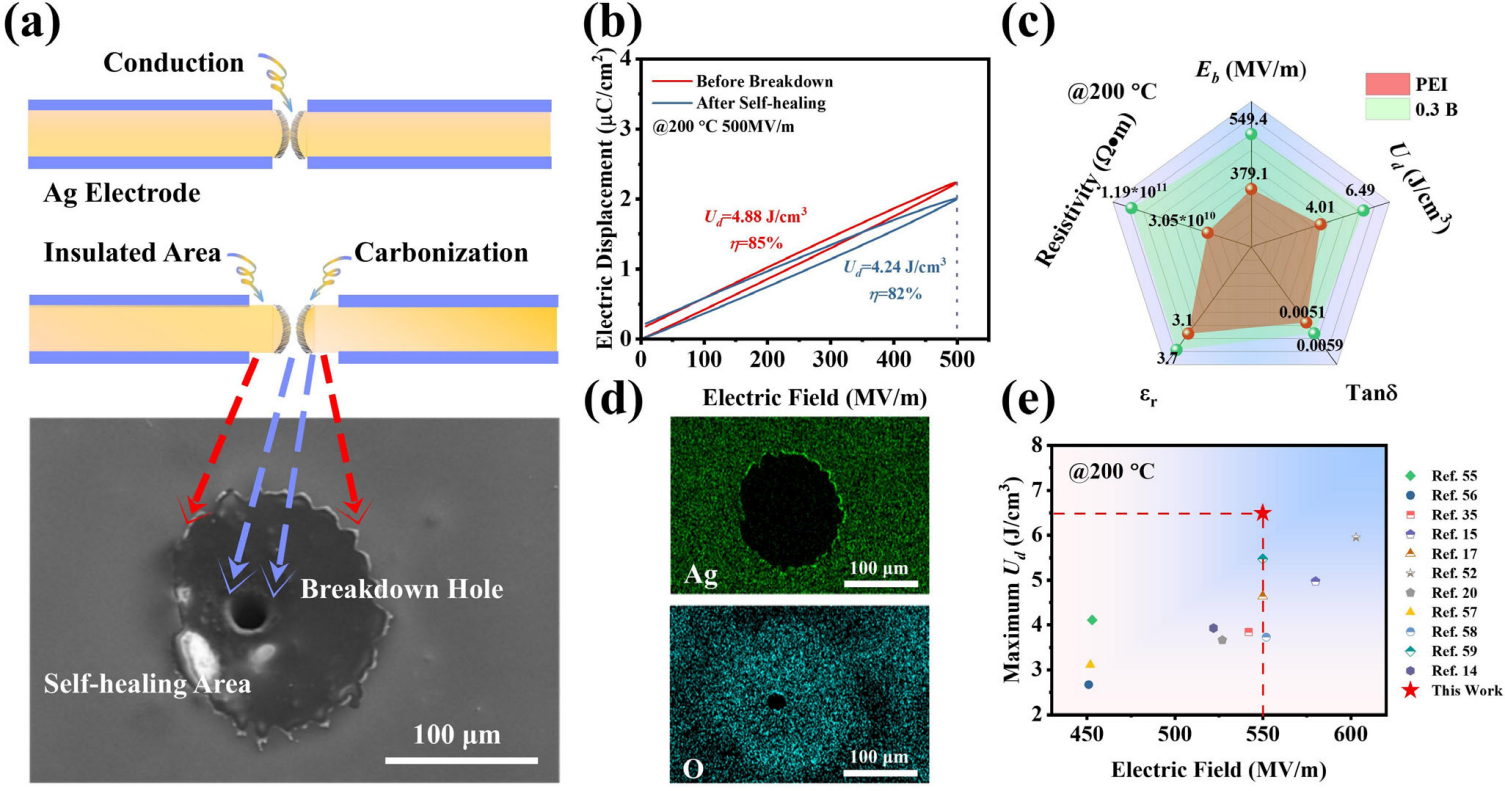
Self‐healing ability analysis and performance comparison of PEI composite films. (a) Behavior of the polymer film after breakdown as well as self‐healing; (b) Energy storage behavior of the film before and after breakdown; (c) Comparison of the performance of PEI and 0.3 B films; (d) Energy spectrum scanning of the film after breakdown; (e) Comparison between the performance of the present work and that of the PEI composite film that has been reported at 200°C.

Unlike ceramic capacitors, polymer film capacitors not only have rapid charging and discharging speeds, but also have their own unique self‐healing capabilities [53]. One of the causes of failure of polymer films when operating at high temperatures is breakdown failure due to high voltage. At the moment of failure, the higher temperatures due to partial discharges cause localized carbonization of the polymer film, resulting in conductive pathways (Figure 6a). However, owing to its unique self‐healing capability, when faults occur under conditions of high temperature and high electric fields, theoretically localized temperatures exceeding 660°C would simultaneously be generated, causing the vaporization of electrodes [54]. This consequently exposes additional areas of polymer insulation, thereby further suppressing the current. The energy storage behavior of the 0.3 B film before and after breakdown is given in Figure 6b, and its energy storage density and η are 4.88 J cm^−3^ and 85% before self‐healing and 4.24 J cm^−3^ and 82% after self‐healing at 500 MV m^−1^. It is shown that the film still maintains a good energy storage behavior after experiencing breakdown, which greatly improves its lifetime. Lastly, the performance of the PEI composite film at different high temperatures was compared with that of previously reported PEI composite films. It is known that the 0.3 B film has improved energy storage capacity, which is also mainly due to the introduction of BNNDs effectively trapping the charge and weakening the conjugation effect, which reduces the energy losses and improves the η of the composite film. In conclusion, the film exhibits a high *U_d_* (Figure 6e; Figure S36b), which greatly contributes to the potential application of the film [14, 15, 17, 20, 31, 52, 55, 56, 57, 58, 59].

## 3 Conclusion

In this study, BNNDs with wide band gaps were first prepared and then introduced into PEI films. Due to the interfacial polarization and the microcapacitive structure formed inside, the dielectric constant of the composite film is increased to 4.3 at 10^3^ Hz. The wide band gap characteristic of BNNDs enables effective charge confinement in the composite films under extreme operational conditions. When it reaches saturation, it produces a certain repulsive effect on the charge, further prolonging the movement path of the charge, thus reducing the energy losses. Alternatively, the introduction of BNNDs further weakens the conjugation effect between the chains of the PEI molecules, thus further inhibiting the charge transport. As a result, the high‐temperature discharge energy density of the PEI composite membrane is achieved. At 150°C, ultrahigh *U_d_* of 7.90 J cm^−3^ and η of 79% are realized. And high *U_d_* of 6.49 J cm^−3^ and η of 65% are also achieved at 200°C. In addition, the composite film has good self‐healing ability with *U_d_* of 4.88 and 4.24 J cm^−3^ before and after self‐healing at 500 MV m^−1^, respectively. Ultimately, the film has an increased pulse power density as well as reduced heat as a capacitor, which greatly increases its potential for application. The present work provides an ideal design for the preparation of polymer dielectric films with high temperature resistance.

## Conflicts of Interest

The authors declare no conflicts of interest.

## Supporting information




**Supporting File**: advs73797‐sup‐0001‐SuppMat.docx.

## Data Availability

The data that support the findings of this study are available from the corresponding author upon reasonable request.
