# Supplementary material for: Wide Band Gap Boron Nitride Nanodots‐Incorporated Polyetherimide Dielectrics for High‐Temperature Dielectric Energy Storage
Source: Adv Sci (Weinh). 2026 Jan 12;13(17):e20484. doi: 10.1002/advs.202520484 (PMC13042593; doi:10.1002/advs.202520484)
Supplement: Supplementary file 1 — Supporting File: advs73797‐sup‐0001‐SuppMat.docx. [file ADVS-13-e20484-s001.docx]

**Supporting Information**

**Wide Band Gap Boron Nitride Nanodots-Incorporated Polyetherimide Dielectrics for High-Temperature Dielectric Energy Storage**

Wen-jin Hu^1^, Wen-hao Huang^1^, Nan Zhang^1^, De-xiang Sun^1^, Yong Wang^1^^[[1]](#footnote-0)^*, Jing-hui Yang^1^*

^1^Key Laboratory of Advanced Technologies of Materials (Ministry of Education), School of Chemistry, Southwest Jiaotong University, Chengdu, 610031, China

* Corresponding author

E-mail: yangjinghui@swjtu.edu.cn (J.H. Yang), yongwang1976@swjtu.edu.cn (Y. Wang).

**Experimental Section**

**1. Materials**

*N*-Methylpyrrolidone (NMP) was purchased from Chengdu Cologne Co, Ltd (AR, ≥99%). PEI pellets purchased from SABIC (PEI, Ultem1000, $M_{w}$=50,000 g mol^-1^). Melamine was purchased from Aladdin (99%). Boric acid purchased from Aladdin (AR, ≥99.5%). All chemical reagents were used as received without further purification in the materials preparation process.

**2. Preparation of BNNDs**

Firstly, 2 g of boric acid and 0.3 g of melamine were taken and dissolved in deionized water. The precursor solution was transferred to a Teflon-lined autoclave and subjected to hydrothermal treatment at 200 °C for 10 h to synthesize BNNDs. The resulting product was then centrifuged (12,000 rpm, 30 min) and the supernatant was purified through dialysis (MWCO: 3.5 kDa) for 48-72 h. Finally, the resulting solution was freeze-dried to obtain BNNDs.

**3. Preparation of PEI Composite Films**

For the preparation of PEI solution. 1 g of PEI was dissolved in 6 mL of NMP. Subsequently, the mixture was stirred continuously at 50 °C for 12 h to obtain a homogeneous PEI solution. Firstly, different mass fractions of BNNDs as 0.05 wt%, 0.1 wt%, 0.3 wt%, 0.7 wt% and 1 wt% (named as 0.05 B, 0.1 B, 0.3 B, 0.7 B, 1 B, respectively) were weighed and dispersed in 6 mL of NMP. After treating the composite solution in a 400 W ultrasonic bath for 1 h, different masses of PEI particles were added to it and stirred for more than 12 h at 50 °C. All PEI composite solutions are cast onto glass substrates and then coated using a squeegee. Finally, the films were placed in a vacuum oven at 110 °C for 6 h and then baked at 200 °C for 3 h in order to completely remove the solvent. The preparation process of PEI composite films is shown in Figure S1b.

**4. Characterization**

The crystal structure characteristics of PEI films were observed by X-ray diffraction using Panalytical. The operating voltage and current were divided into 45 kV, 40 mA, and the films were fixed on a silicon substrate during testing. For PEI composite films, the scanning range was 10º-30º. For BNNDs, the scanning range was 10º-70º.

The functional groups of the BNNDs and PEI composite films were tested by Fourier Transform Infrared (FTIR) spectroscope using a Nicolet iS20 spectrometer from Thermo Fisher Scientific, where the scanning wavelengths were in the range of 500-4000 cm^-1^ with a step size of 4 cm^-1^.

The morphology of BNNDs and their dispersion within PEI composite films were analyzed using a transmission electron microscope (TEM) JEM-2100F from Japan Electronics Co., Ltd. The voltage employed in the experiment was 200 kV.

The surface micro-morphologies of PEI composite films were observed by model Multimode 8 in atomic force microscope (AFM) in tapping mode. To enable better imaging, the samples were fixed on the substrate while performing the tests.

The specific elemental compositions of PEI composite films and BNNDs were further analyzed by using a Thermo Scientific K-Alpha X-ray photoelectron spectroscope (XPS) instrument from the United States. Al K$\alpha$ ray was used as the excitation source with an operating voltage of 12 kV and a current of 6 mA. Subsequently, the energies of PEIs and BNNDs were further tested using a Thermo SCIENTIFIC Nexsa UV photoelectron spectrometer (UPS) with an energy resolution of ≤ 120 meV.

Microstructural characterizations of the PEI composite films were performed using field-emission scanning electron microscope (SEM) JSM-7800F from JEOL under optimized imaging conditions. In order to prevent charges from accumulating on the sample surface, a gold layer with a thickness of 15 nm was sputtered on the sample surface by an ion sputterer prior to SEM observation. Here, an electron acceleration voltage of 2.7 kV and a working distance of 10 mm were used.

Differential scanning calorimetry (DSC) testing of the composite film was conducted using the DSC-204 instrument from NETZSCH to determine their $T_{g}$. Testing was conducted under a nitrogen atmosphere at a heating rate of 10 ºC min^-1^.

Thermo-mechanical analysis (TMA) of the PEI composite film was performed using a TMA-402 from NETZSCH. The load force was set at 0.1 N. The heating rate was 5 ºC min^-1^.

UV-visible spectroscopic tests were performed on PEI composite films using a SHIMADZU UV-2600 spectrophotometer. The scanning range was 200-800 nm, where the scanning step was 1 nm.

A broadband dielectric meter was used to evaluate the ambient and variable temperature dielectric properties of PEI composite films using Novocontrol's Concept 41 system. For the room temperature tests, the frequency range was 10^3^-10^6^ Hz. Subsequently, the dielectric temperature spectrum of the composite films was tested at different temperatures at 10^3^ Hz. A DC voltage of 1 V was applied during the tests. All samples were plated with silver electrodes of 10 mm diameter and 60 nm thickness using a magnetron sputterer prior to testing.

The $E_{b}$ of PEI composite films was tested using an electrical breakdown instrument, and the test was carried out using a BDJC-50 kV dielectric voltage tester from Beiguang Jingyi Instruments. Prior to the tests, circular silver electrodes with a diameter of 4 mm were plated on the surface of the films. The boost rate was 500 V s^-1^ and the peak leakage current was 2 mA.

The D-E curves and leakage currents of the PEI composite films were evaluated using a Trek 610E ferroelectric test system from the USA. Prior to testing, all composite films were coated with silver electrodes with a diameter of 2 mm and a thickness of 60 nm.

The fluorescence emission spectra of PEI composite films were obtained by photoluminescence (PL) testing using the Edinburgh FLS1000, with an excitation wavelength of 375 nm.

To further evaluate the charge trapping ability of PEI composite films, they were tested for thermally stimulated depolarizing current (TSDC) using an electrostatic meter (KEITHLEY 6517B). The specific test conditions were as follows: firstly, the temperature was increased to 200 ºC and polarized under an electric field of 50 MV m^-1^ for 30 min, then cooled down to 0 ºC at 10 ºC min^-1^ and held for 3 min. Finally, the circuit was short-circuited and heated up to 220 ºC at a rate of 3 ºC min^-1^, while the current change of the circuit was recorded. The obtained TSDC curves were further analyzed for trap depth by the following equations:

$$\begin{aligned} \text{E}\text{=}\frac{\text{2.47}\text{K}_{\text{B}}{\text{T}_{\text{P}}}^{\text{2}}}{\text{∇}\text{T}}\#\left( \text{1} \right) \end{aligned}$$

where $E$ is the trap depth, *K_B_* is the Boltzmann constant, $T_{P}$ is the temperature of the current peak, and $\nabla T$ is the temperature difference of the half peak width. Following this, the trapping of charge by the PEI composite film was further calculated by the following equation:

$$\begin{aligned} \text{Q}\text{=}\left( \frac{\text{60}}{\text{ν}} \right)\int_{\text{T}_{\text{1}}}^{\text{T}_{\text{2}}} \text{I}\left( \text{T} \right)\text{dT}\#\left( \text{2} \right) \end{aligned}$$

where $Q$ is the amount of charge captured by the film, and $\text{ν}$ is the heating rate during the depolarization phase, which is 3 ºC min^-1^ in this test. $T_{1}$ is the starting temperature of the peak current, and $T_{2}$ is the ending temperature of the peak current.

**5. Theoretical Calculation**

Firstly, the electrostatic potentials of PEI and BNNDs were calculated using the Dmol3 module in Materials Studio (MS). The electrostatic potential distribution of PEI and BNNDs was obtained by optimizing the structure and energy system. In order to further verify the free volume variation of the composite films, the free volumes of PEI and 0.3 B films were calculated using the Forcite module. Specifically, the two systems were first structurally optimized, followed by an annealing module to further relax their structures with higher internal energies at annealing temperatures of 300 K-500 K in steps of 5. Finally, the obtained structures were calculated by NVE of molecular dynamics.

To investigate the interaction between BNNDS and PEI, the two molecular structural models were simulated using Gaussian software. First of all, simple structural optimization was performed on PEI, BNNDS, PEI-PEI, and PEI-BNNDS using the PM6 semi-empirical method. Then, structural optimization and energy calculations were performed on them using the B3LYP/6-31g(d) basis set and density functional. At the same time, to ensure computational accuracy, the basis set superposition error (BSSE) was rigorously corrected during interaction energy calculations using the counterpoise method. The binding energy was evaluated as:

$$\begin{aligned} E(Interaction)=E(AB\_Corrected)-E(A)-E(B)\#\left( 3 \right) \end{aligned}$$

Where E(Interaction) denotes interaction energy between two molecules , E(AB_Corrected) is the total energy of the composite molecular structure, E(A) and E(B) are the energies of a single molecule. To further visualize the interaction between PEI and BNNDs, a density reduced gradient (RDG) analysis was performed using the Multiwfn program, identifying steric effects, van der Waals interactions. The resulting spatial distribution of weak interactions was visualized using VMD with isosurface rendering.

1. **Phase Field Simulation**

The breakdown paths inside the composite film were analyzed using comsol software. Weak form partial differential equations are used for the calculations. For better calculation, first the total free energy function of the system is as follows:

$$\begin{aligned} \prod\left[ s,\emptyset\right]=\int_{Ω} \left[ W_{es}\left( E,s \right)+W_{d}\left( s \right)+W_{i}\left( \nabla s \right) \right]dV\#\left( 4 \right) \end{aligned}$$

where $W_{es}(E, s)$ is the electrostatic energy density stored per unit volume of medium and $W_{d} (s)$ is the energy density of damage. $W_{i}(\nabla s)$ is the gradient energy term. $f(s)$ is an interpolating function with respect to the phase field variables, $f(s)$=4$s^{3}$-3$s^{4}$, where $f(0)$=$f(1)$=1, $f’(0)$=$f’(1)$=0. In addition, under the action of an electric field, the dielectric constant is a function of the phase field variable:

$$\begin{aligned} \varepsilon\left( s \right)=\frac{\varepsilon_{0}}{f\left( s \right)+\partial}\#\left( 5 \right) \end{aligned}$$

where $\varepsilon_{0}$ is the dielectric constant of the material. When breakdown occurs, $\partial$=10^-3^ s.

1. **Simulation of Heat Generation Inside a Capacitor**

For film capacitors, the heat generated when they operate under electric field cannot be ignored. Here, by constructing a capacitor with a diameter of 20 mm and a height of 20 mm, metal electrodes and dielectric films were wound alternately. The thickness of the metal electrode is 60 nm. The heat transfer process of the whole model is mainly controlled by the following equations:

$$\begin{aligned} \rho C\frac{\partial T(\mathbf{r})}{\partial t}=\kappa\nabla^{2}T(\mathbf{r})+\sigma(\mathbf{r},T)E^{2}\#\left( 6 \right) \end{aligned}$$

where, $\rho$ is the density, $C$ is the heat capacity of the material, $k$ is the thermal conductivity, $T$ is the temperature, $\sigma$ is the electrical conductivity, and $E$ is the electric field.

1. **Schottky emission conduction**

The Schottky emission conduction can be fitted using the following formula:

$$\begin{aligned} J=AT^{2}\exp(-\frac{\mu_{S}}{kT})exp(\frac{\sqrt{e^{3}/4\pi\varepsilon_{0}\varepsilon_{r}}\sqrt{E}}{kT})\#\left( 7 \right) \end{aligned}$$

where $J$ is the leakage current density of the PEI composite films, *A* is the Richardson constant, $T$ is the test temperature, $\mu_{S}$ is the barrier height, $k$ is the Boltzmann constant, $e$ is the electron charge, $\varepsilon_{0}$ is the vacuum permittivity, $\varepsilon_{r}$ is the dielectric constant of the PEI composite films, and $E$ is the test electric field.

1. **Poole-Frenkel emission conduction**

The Poole-Frenkel emission conduction can be fitted using the following formula:

$$\begin{aligned} J=\sigma_{0}E\exp(-\frac{\mu_{PF}}{kT})exp(\frac{\sqrt{e^{3}/\pi\varepsilon_{0}\varepsilon_{r}}\sqrt{E}}{kT})\#\left( 8 \right) \end{aligned}$$

where $J$ is the leakage current density of the PEI composite films, $\sigma_{0}$ is the conductivity under low electric field, $E$ is the test electric field, $\mu_{PF}$ is the barrier height, $k$ is the Boltzmann constant, $T$ is the test temperature, $e$ is the electron charge, $\varepsilon_{0}$ is the vacuum dielectric constant, and $\varepsilon_{r}$ is the dielectric constant of the PEI composite films.


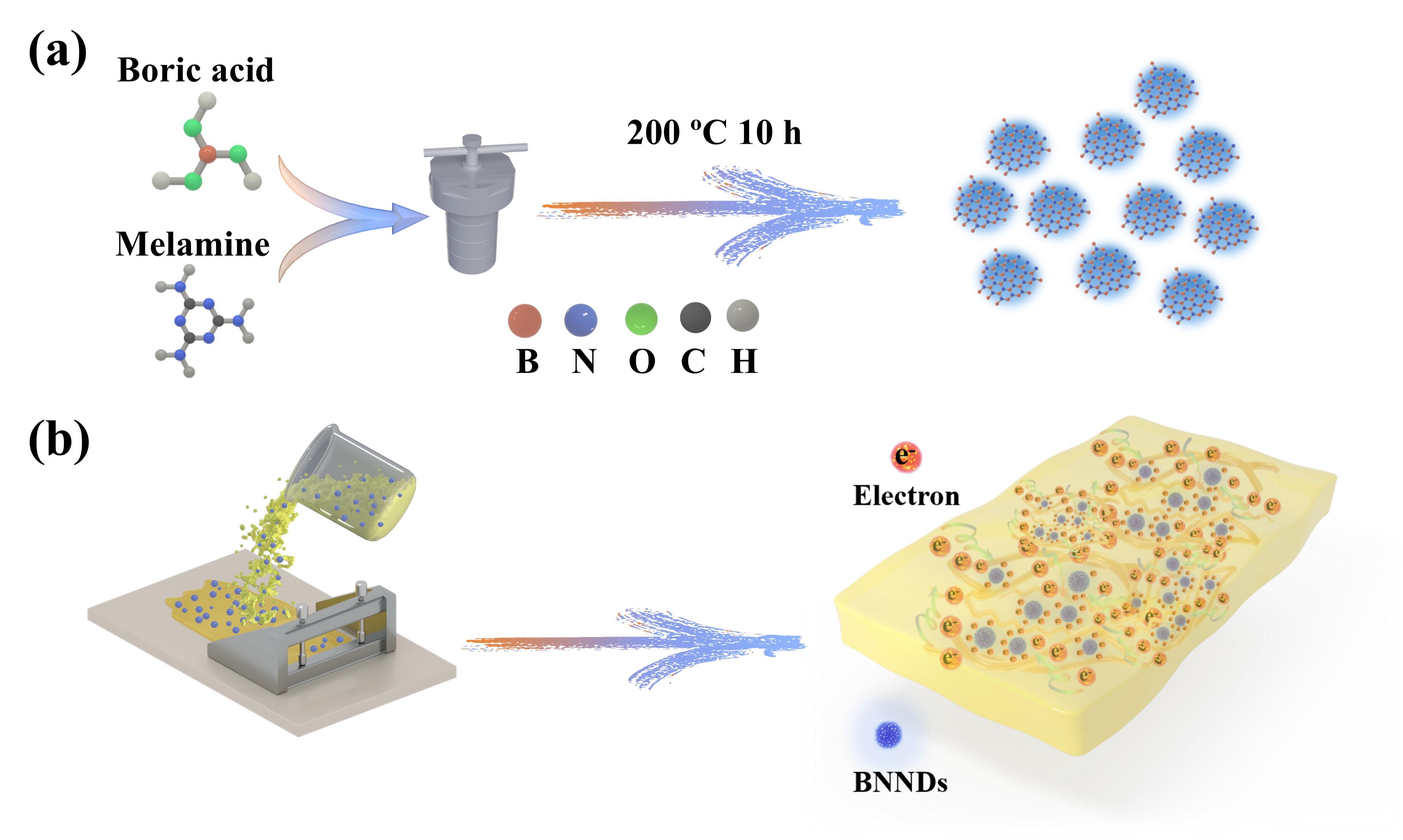


**Figure S1.** (a) Preparation flow chart of BNNDs; (b) Process for the preparation of PEI composite films.


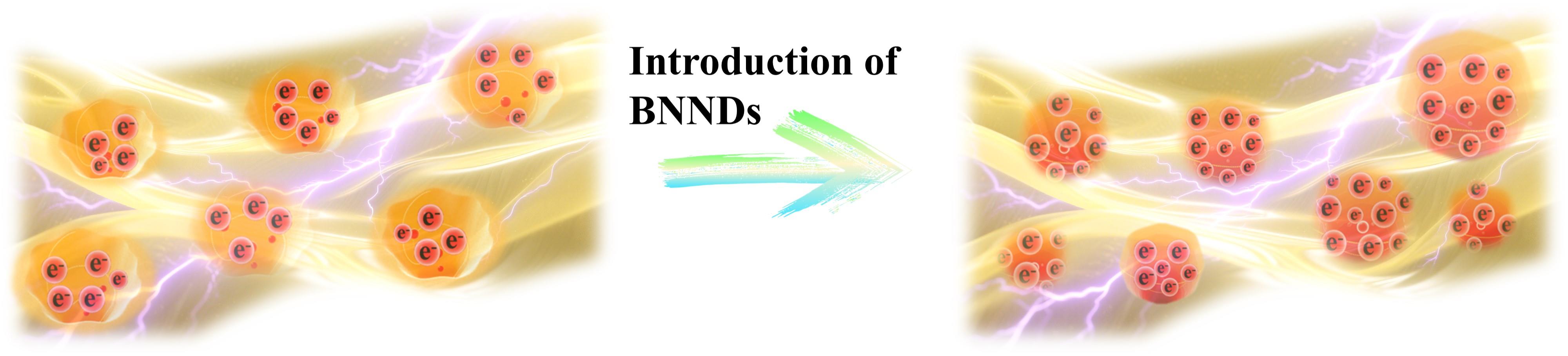


**Figure S2.** Mechanism diagram of charge capture in PEI composite films.


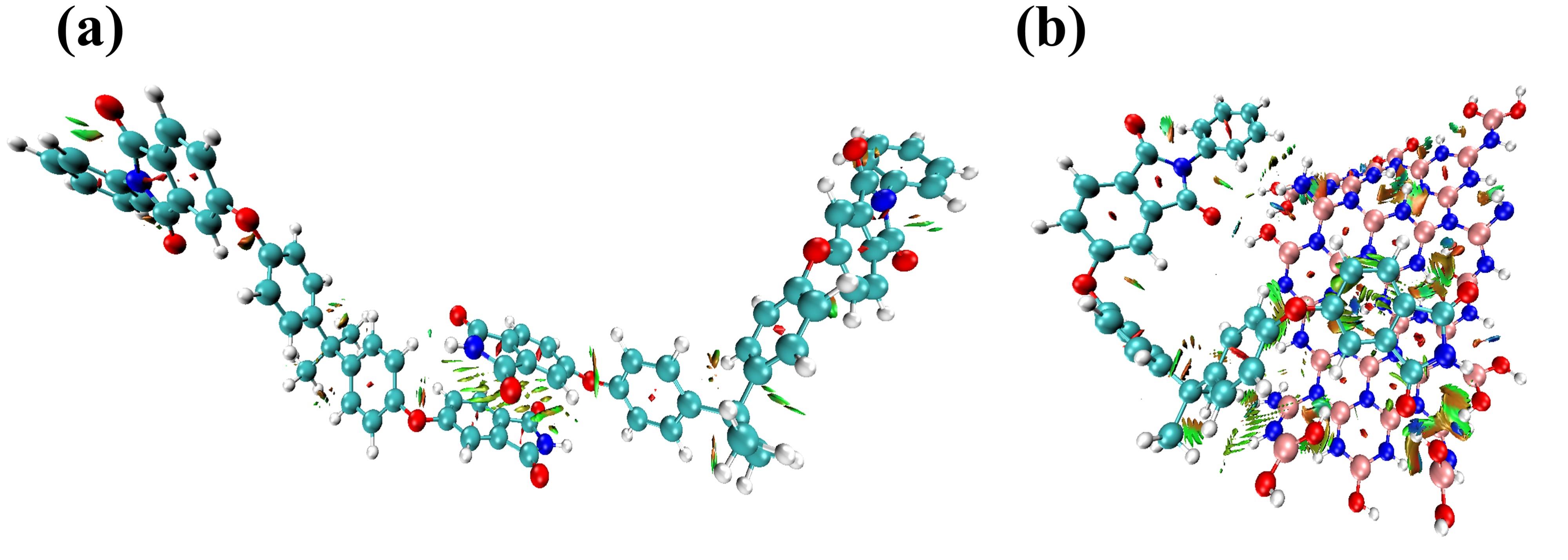


**Figure S3.** Visualization of interactions between PEI and BNNDs. (a) PEI-PEI; (b) BNNDs-PEI.


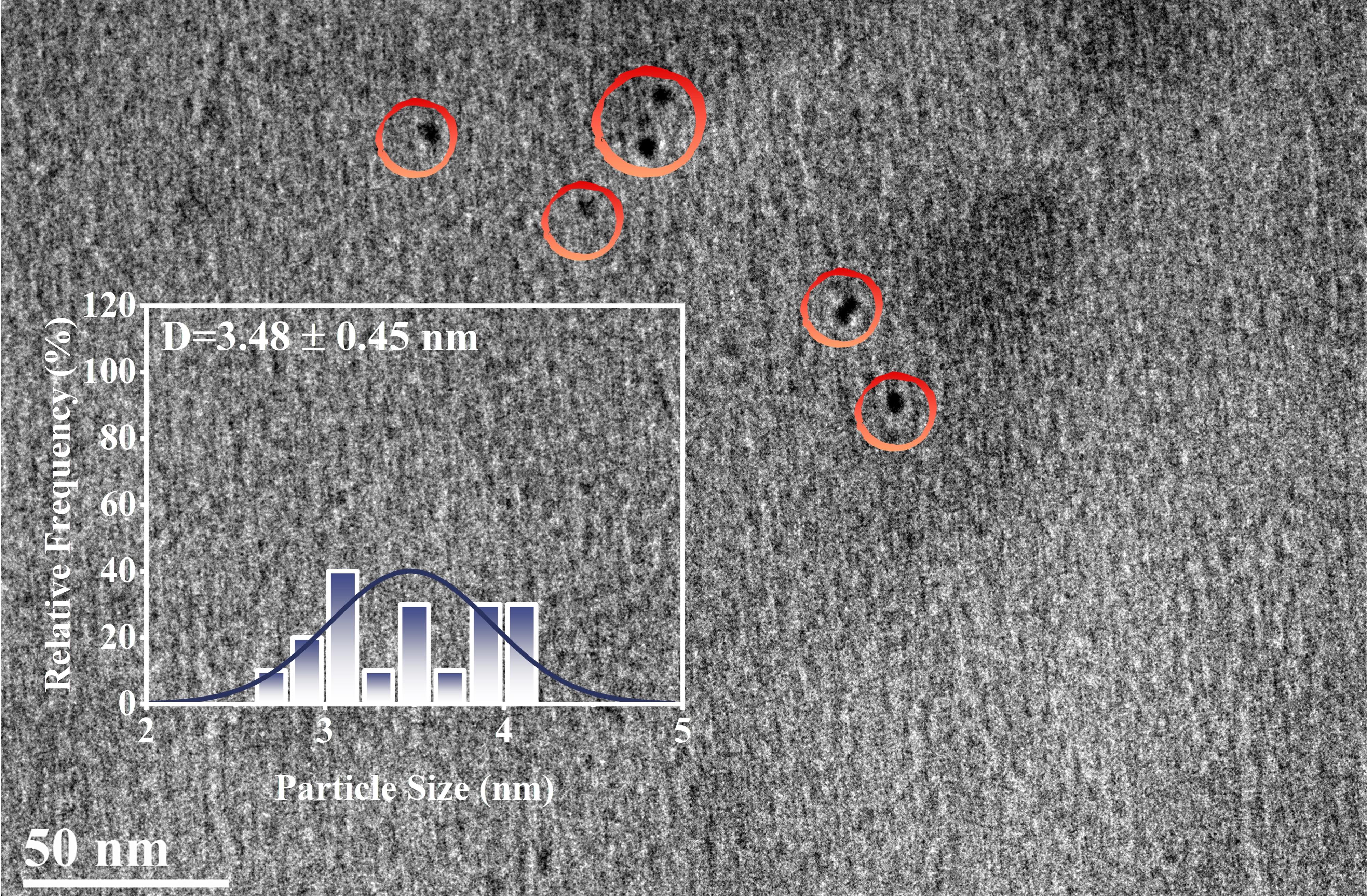


**Figure S4.** TEM image of BNNDs.


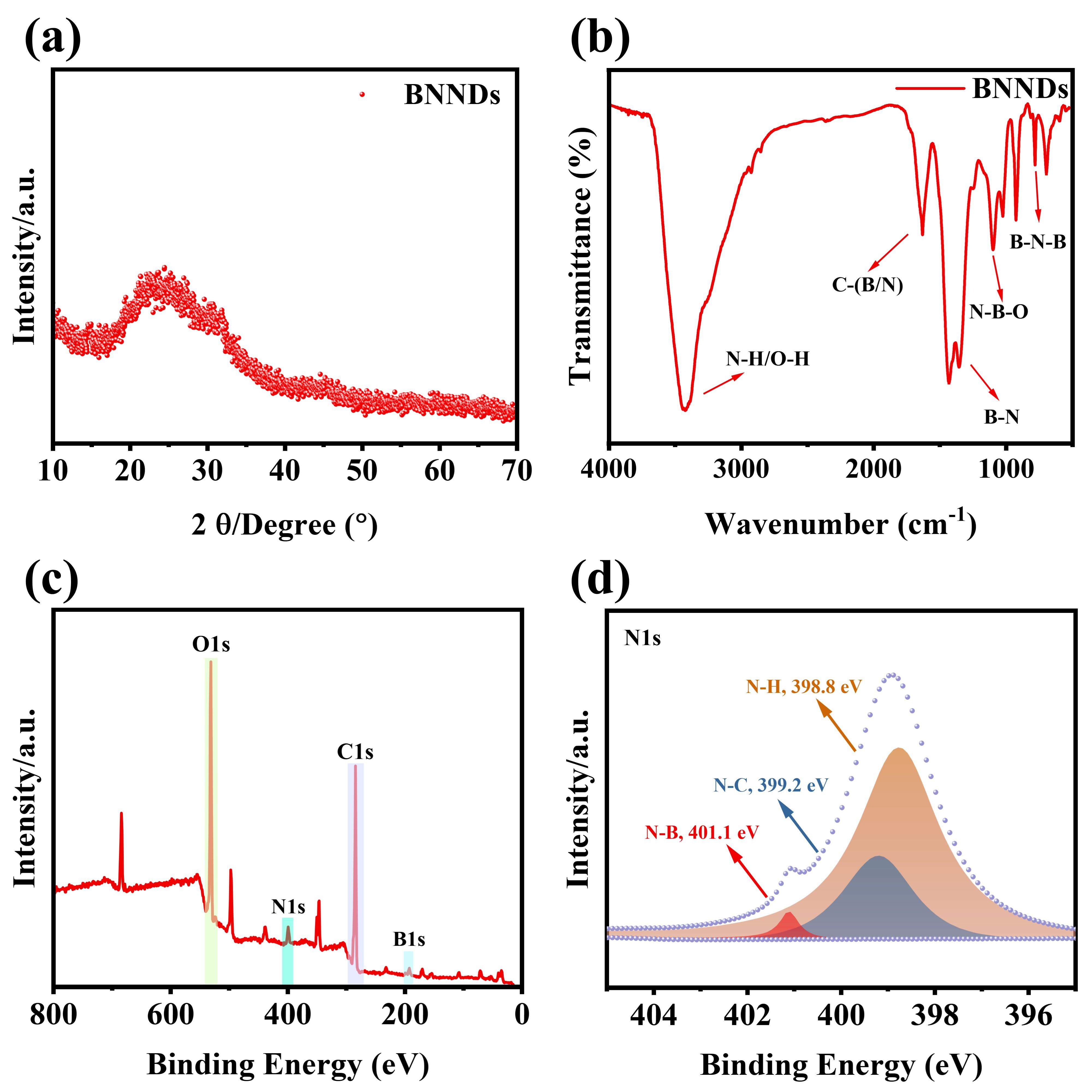


**Figure S5.** Structural characterization of BNNDs. (a) XRD curve of BNNDs; (b) FTIR spectrum of BNNDs; (c) XPS spectrum of BNNDs; (d) N1s high-resolution fine spectra of BNNDs.


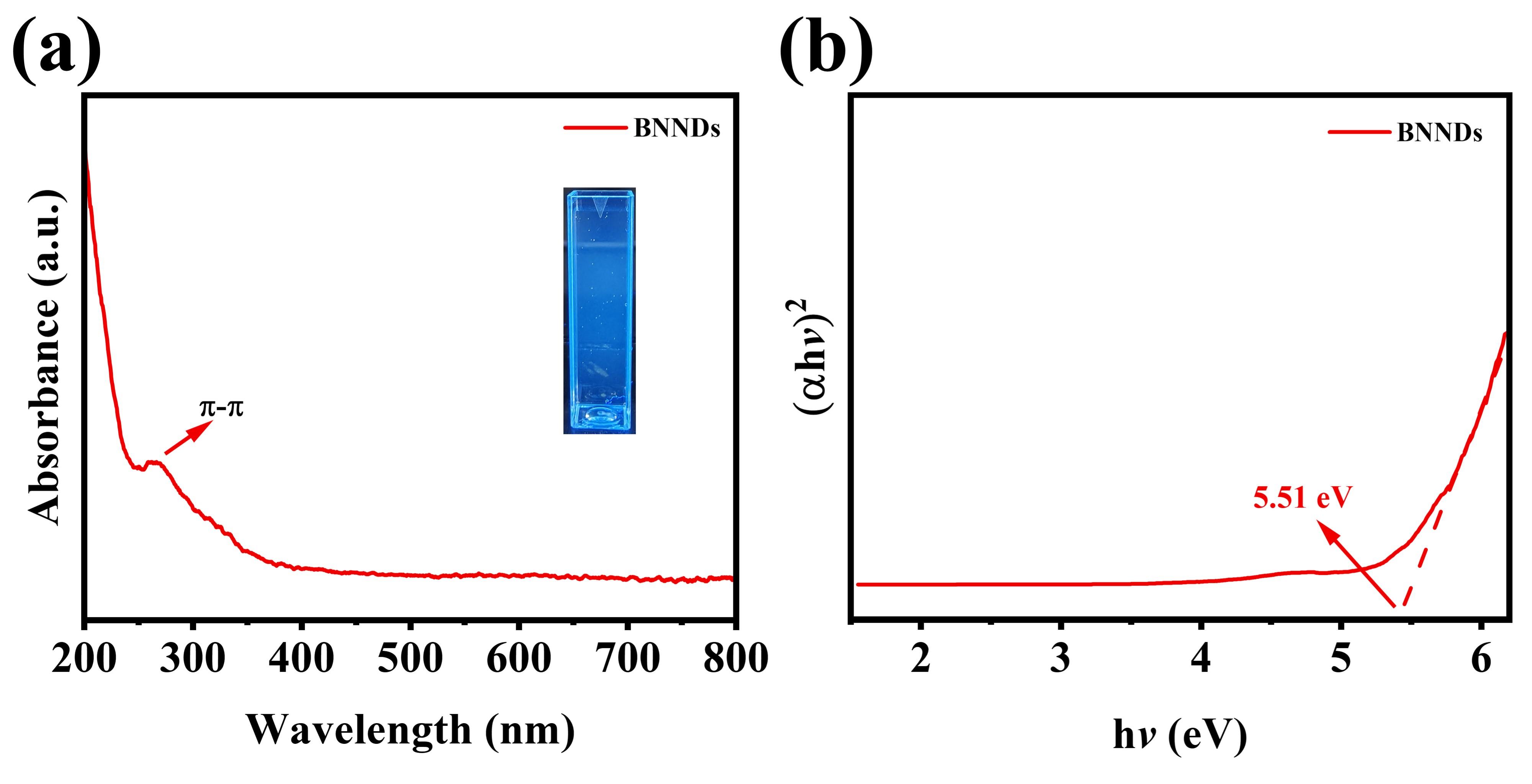


**Figure S6.** Optical band gap analysis of BNNDs. (a) UV-vis spectrum; (b) Band gap calculation of BNNDs.


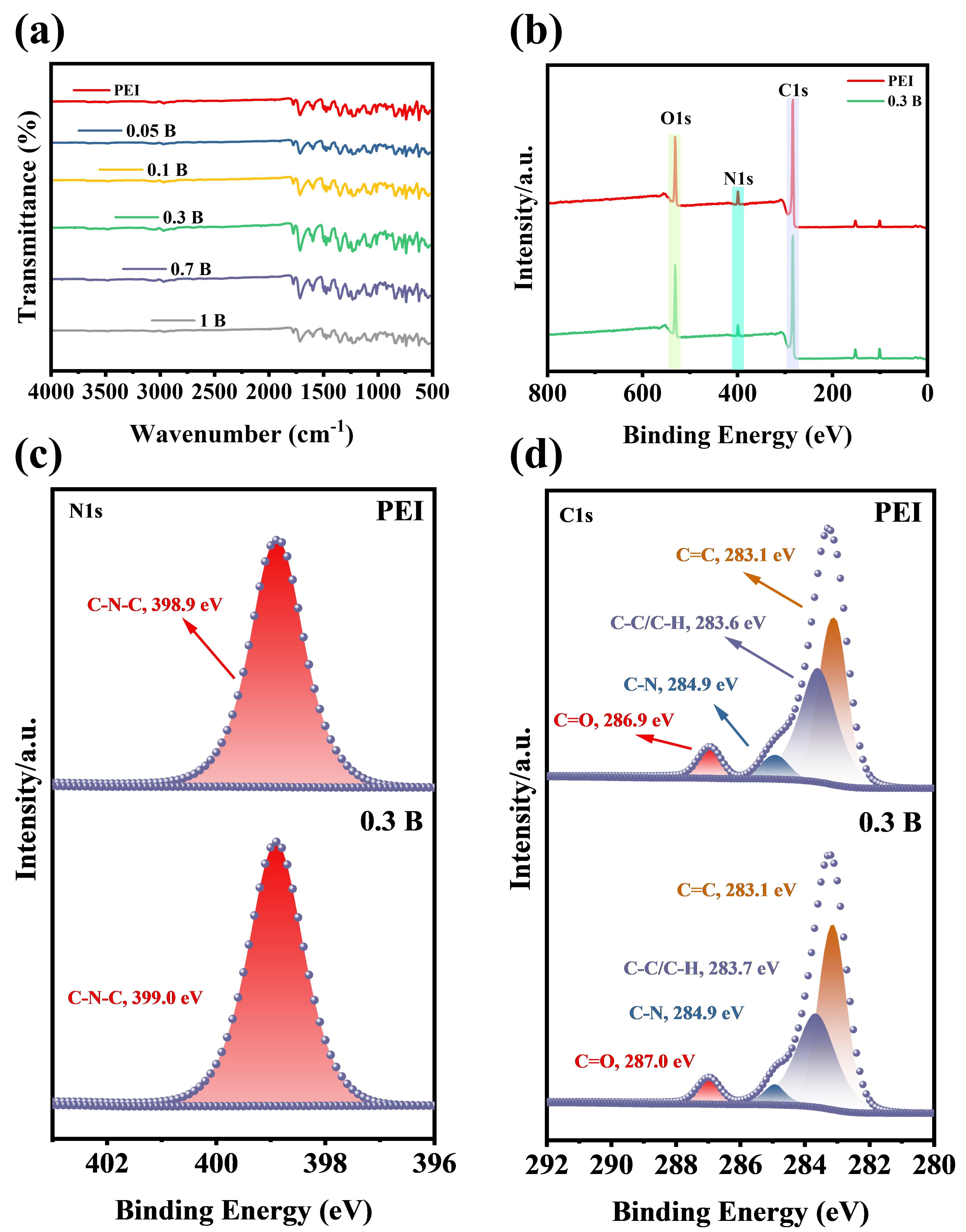


**Figure S7.** Structural characterization of PEI composite films. (a) ATR-FTIR spectra of PEI composite films; (b) XPS gross spectra of PEI and 0.3 B films; (c) N1s high-resolution fine spectrum of PEI and 0.3 B films; (d) C1s high-resolution fine spectra of PEI and 0.3 B films.


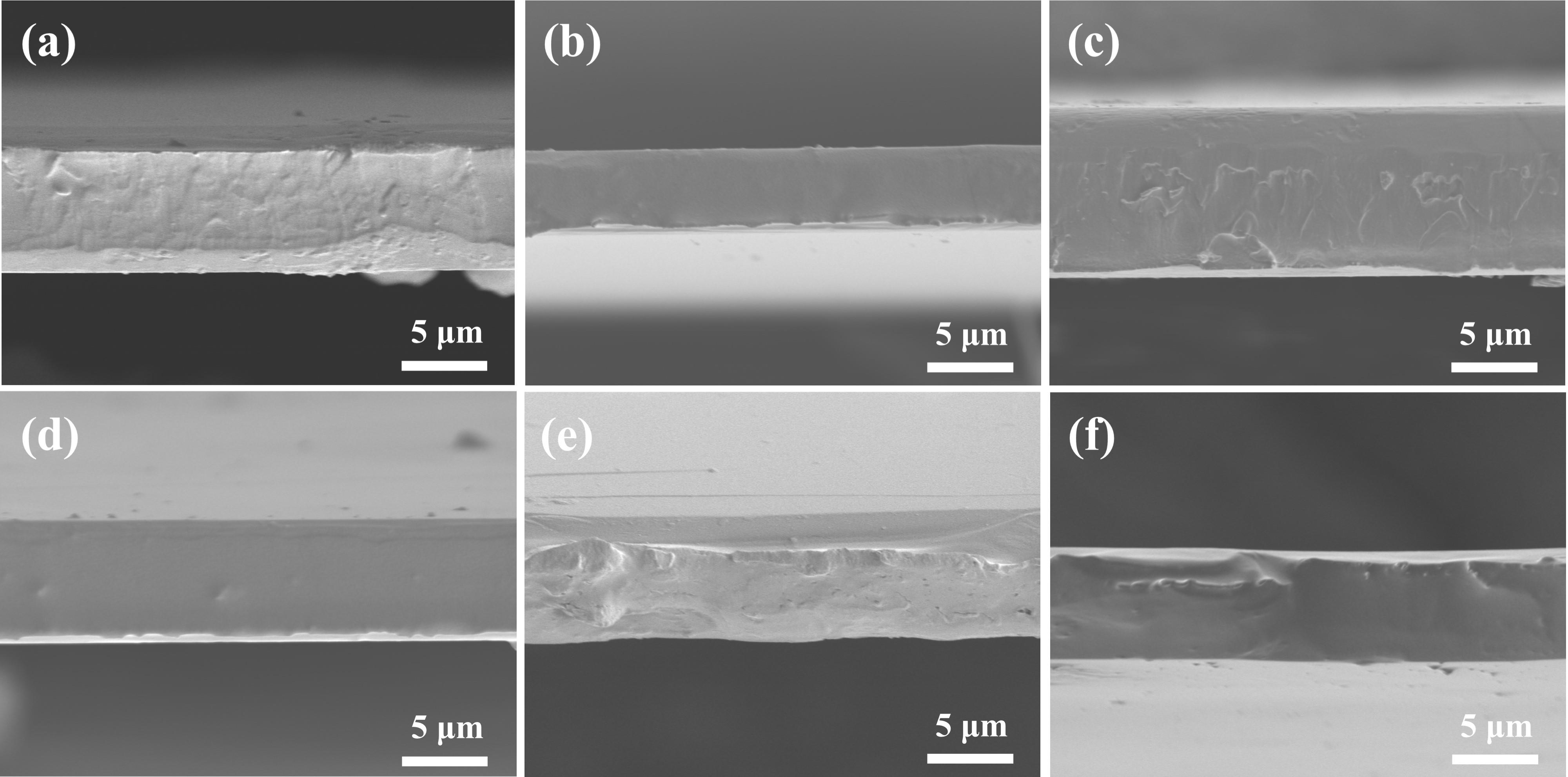


**Figure S8.** Cross-sectional SEM images of PEI composite films. (a) PEI; (b) 0.05 B; (c) 0.1 B; (d) 0.3 B; (e) 0.7 B; (f) 1 B.


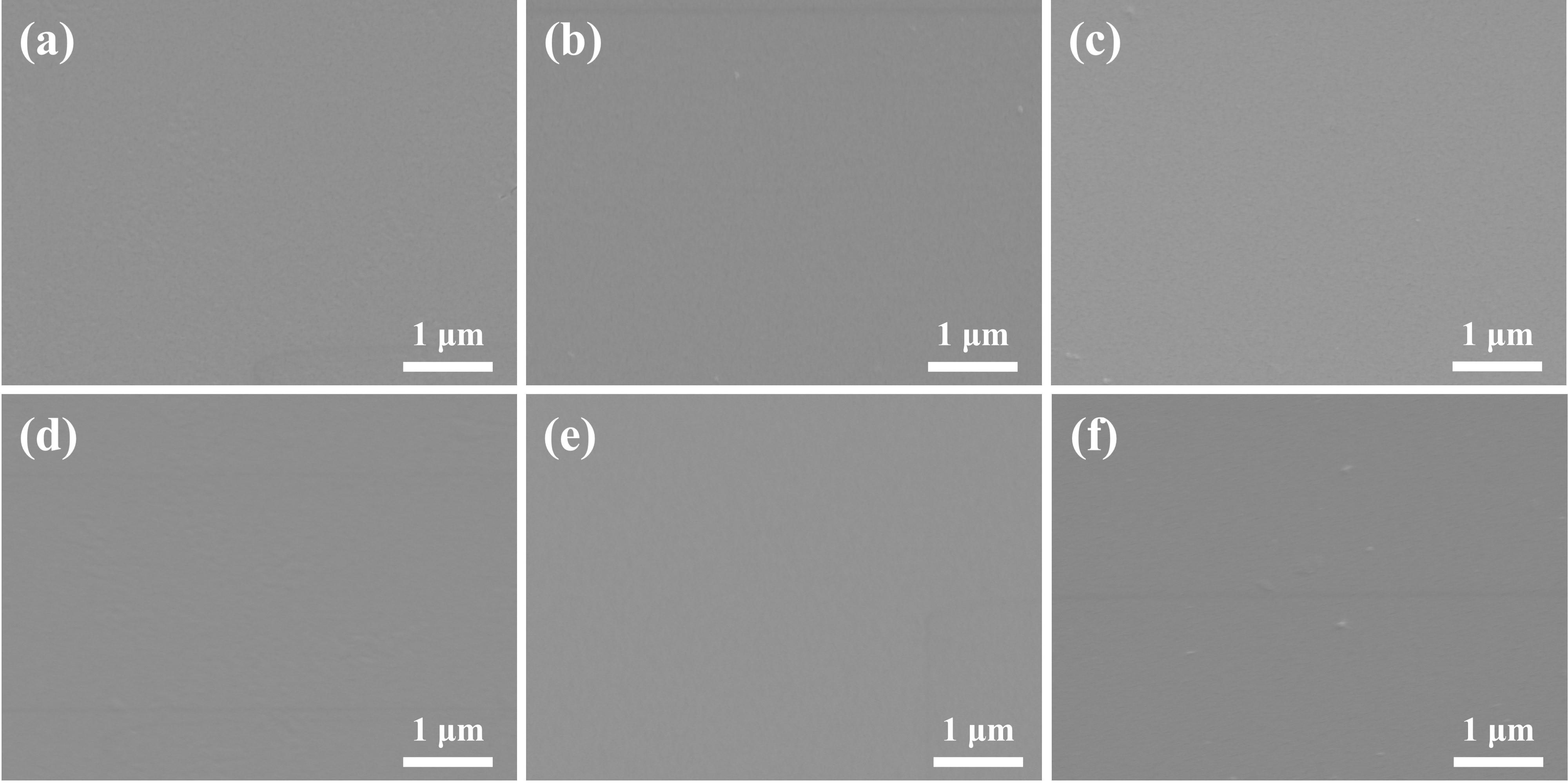


**Figure S9.** Surface SEM images of PEI composite films. (a) PEI; (b) 0.05 B; (c) 0.1 B; (d) 0.3 B; (e) 0.7 B; (f) 1 B.


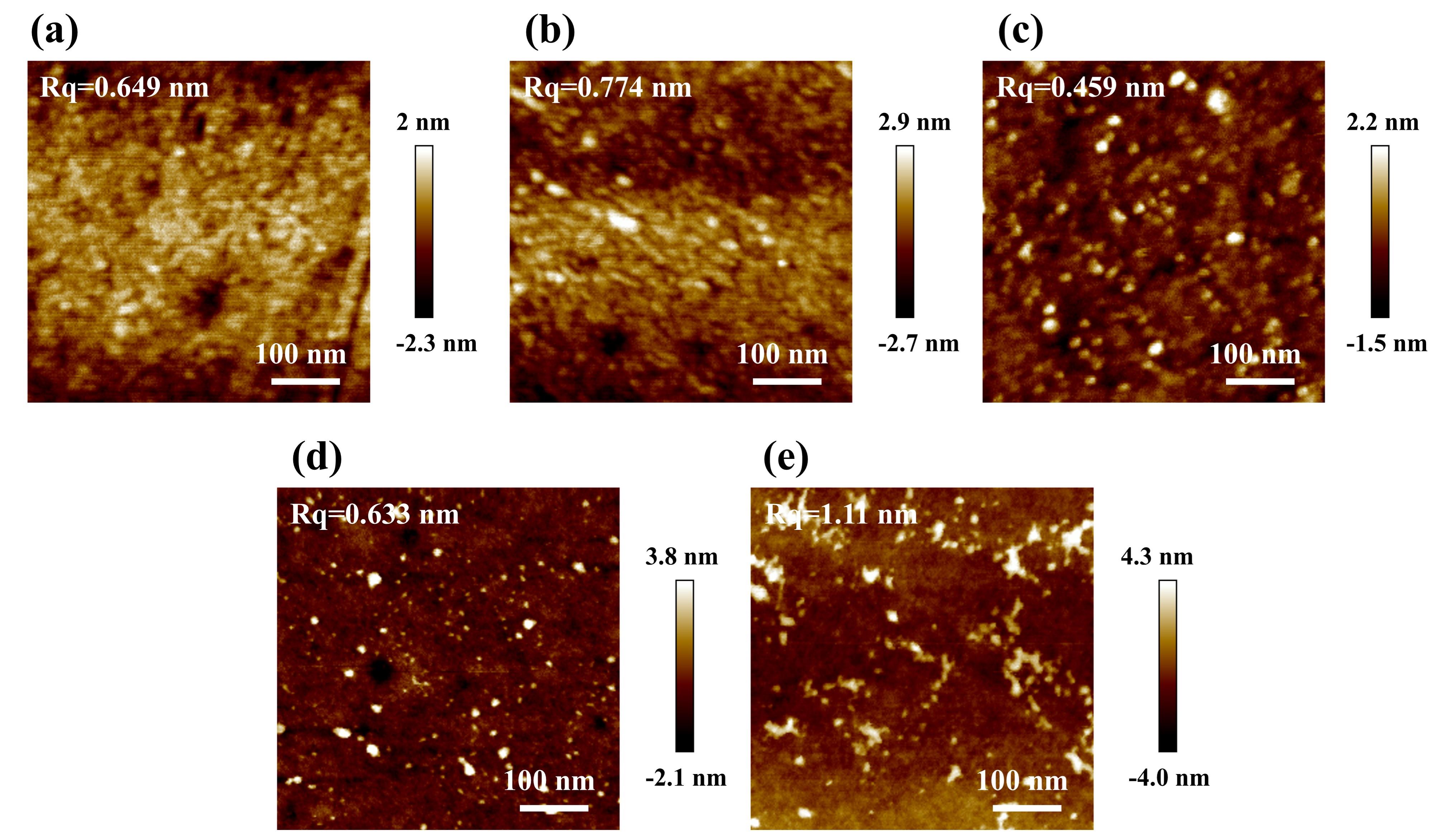


**Figure S10.** Surface AFM images of PEI composite films. (a) PEI; (b) 0.05 B; (c) 0.1 B; (d) 0.7 B; (e) 1 B.


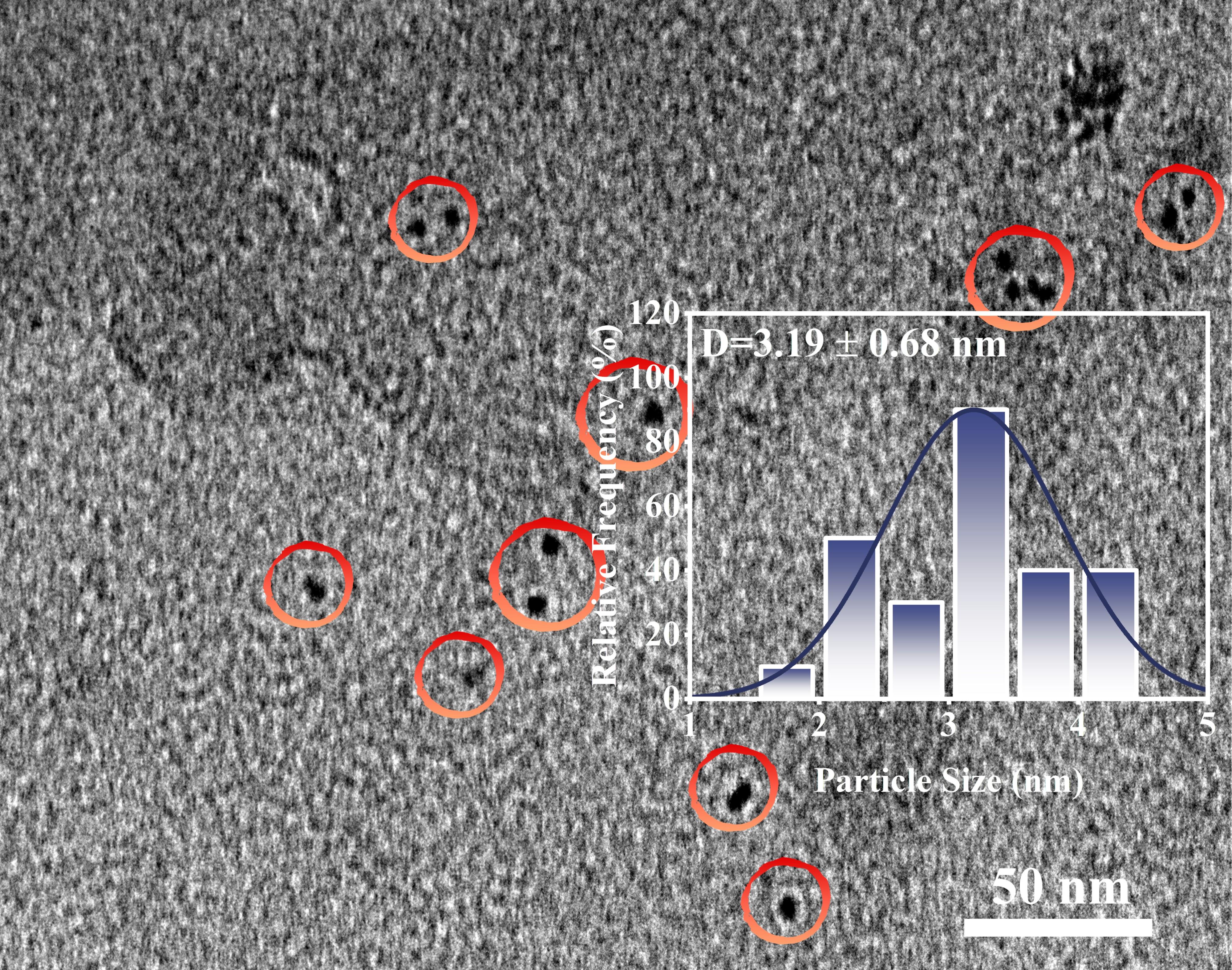


**Figure S11.** TEM image of BNNDs in the film.


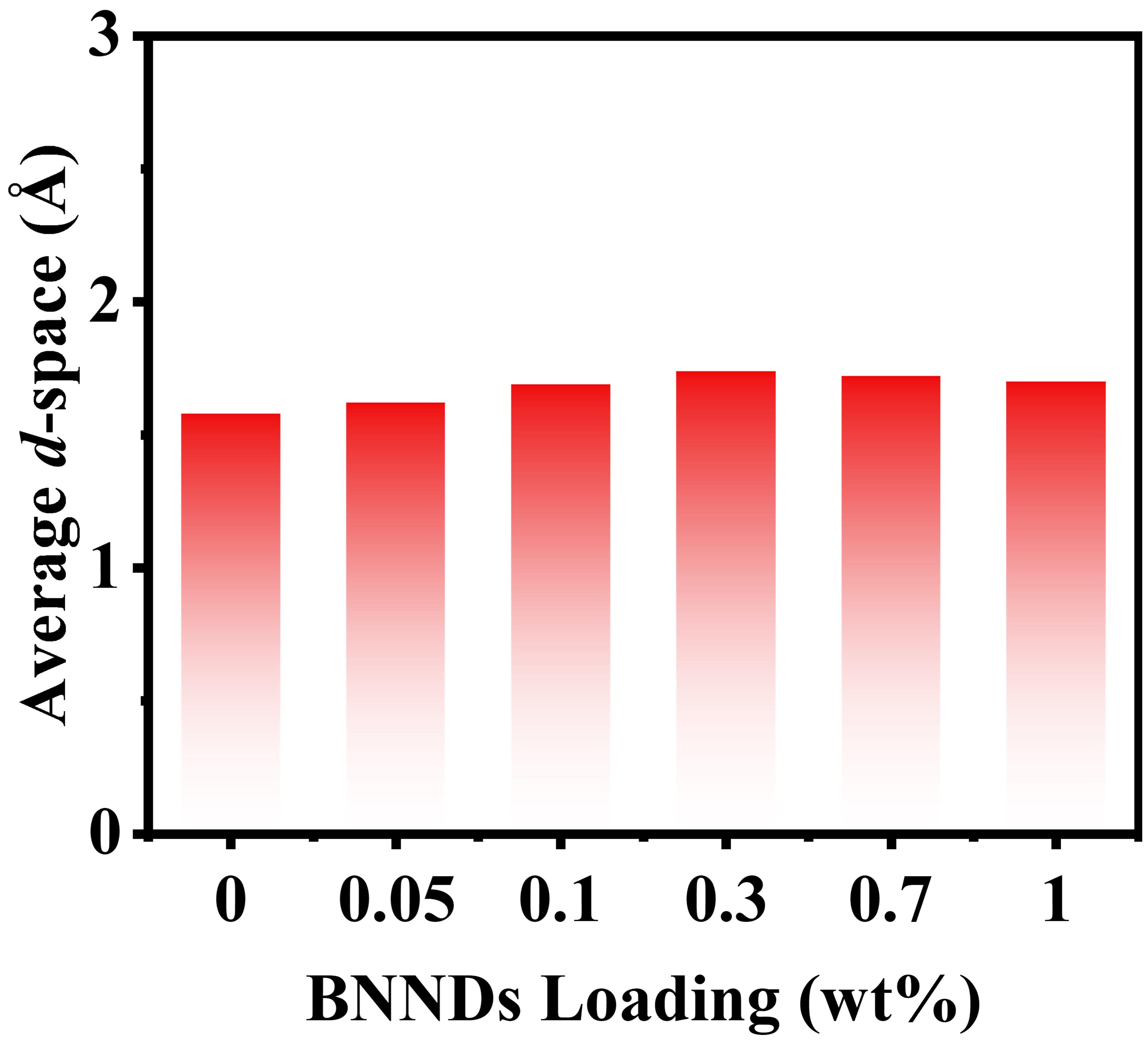


**Figure S12.** Molecular chain spacing of PEI composite films calculated from Bragg's law [1]. ($n\lambda$ =$2dsin\theta$).


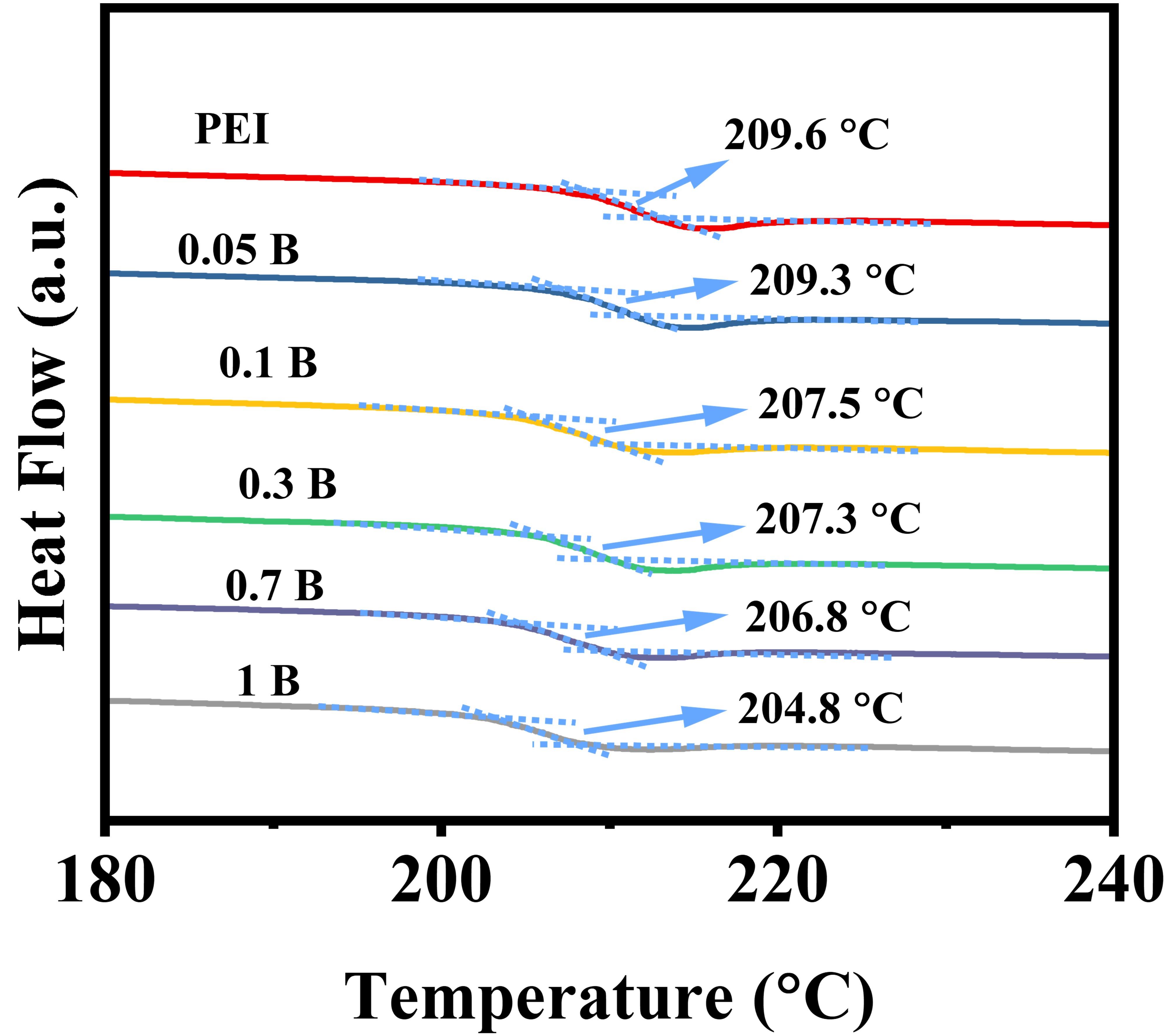


**Figure S13.** Temperature-dependent DSC curves of PEI composite film.


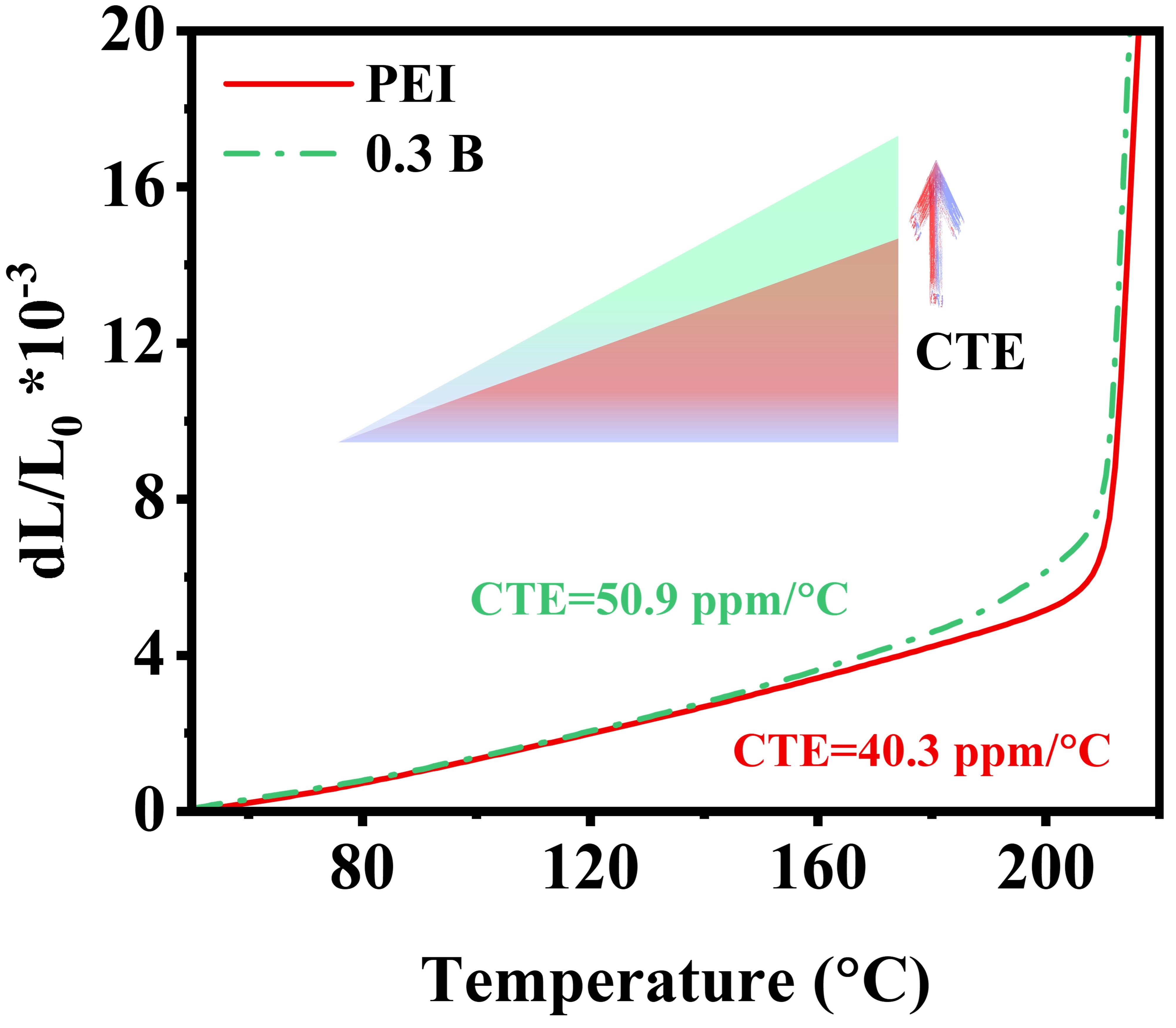


**Figure S14.** TMA curves for PEI and 0.3 B films.


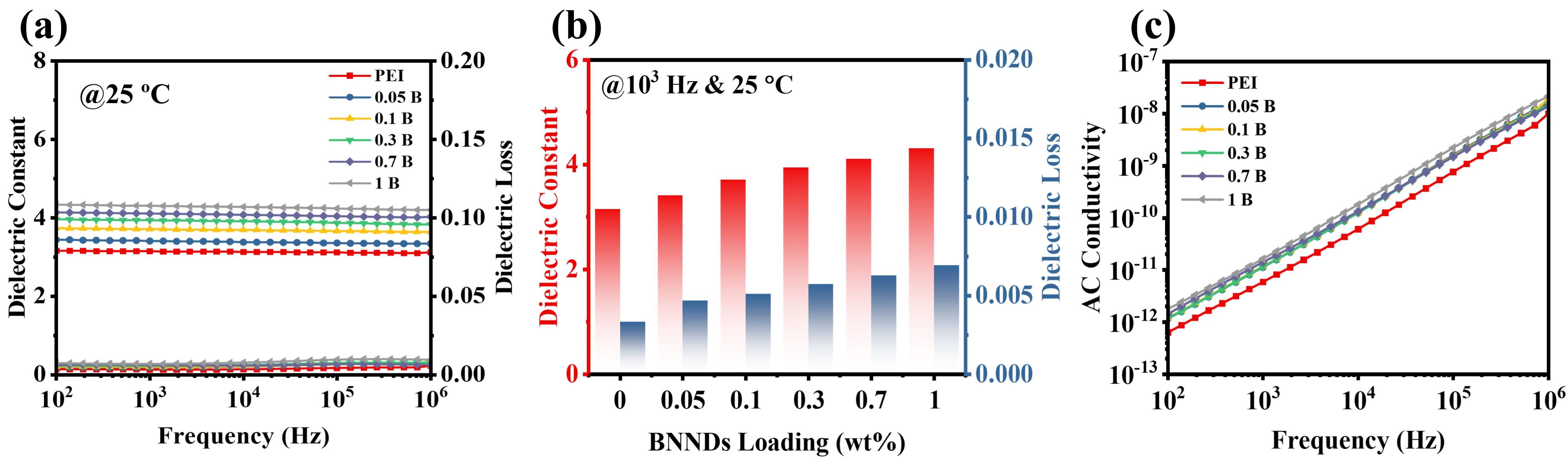


**Figure S15.** Analysis of dielectric properties of PEI composite films. (a) Dielectric constant and tan$\text{δ}$ of PEI composite films at room temperature; (b) Dielectric constant and tan$\delta$ of PEI composite films at 10^3^ Hz; (c) Frequency-dependent conductivity of PEI composite films.


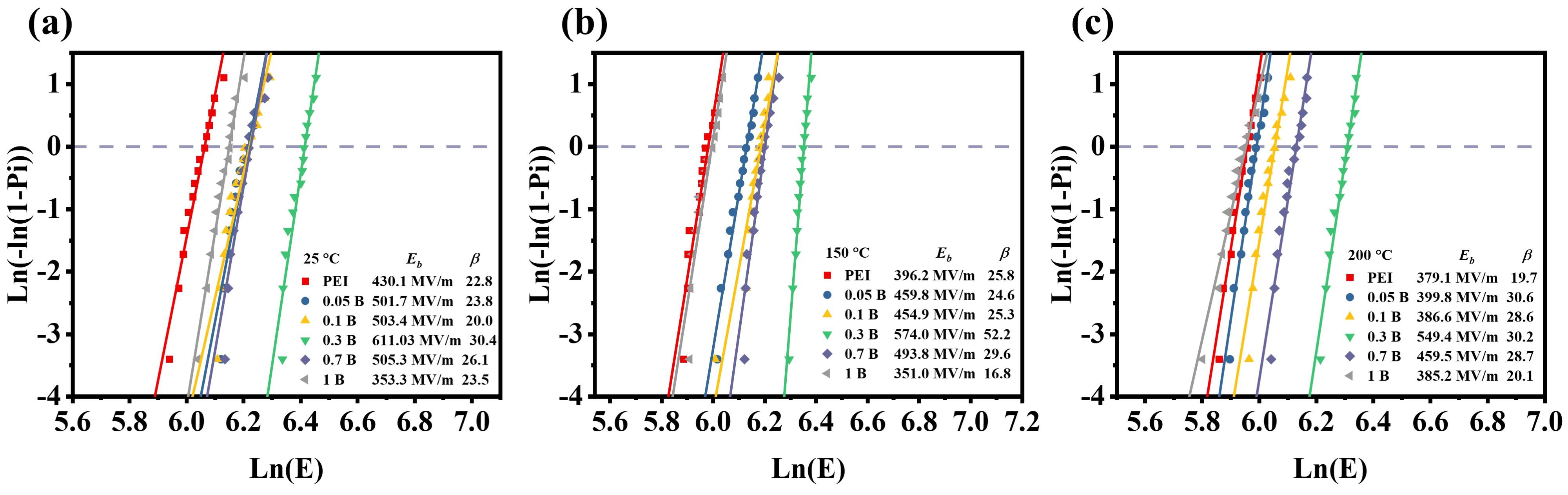


**Figure S16.** Weibull linear fits of PEI composite films at different temperatures. (a) 25 ºC; (b) 150 ºC; (c) 200 ºC.


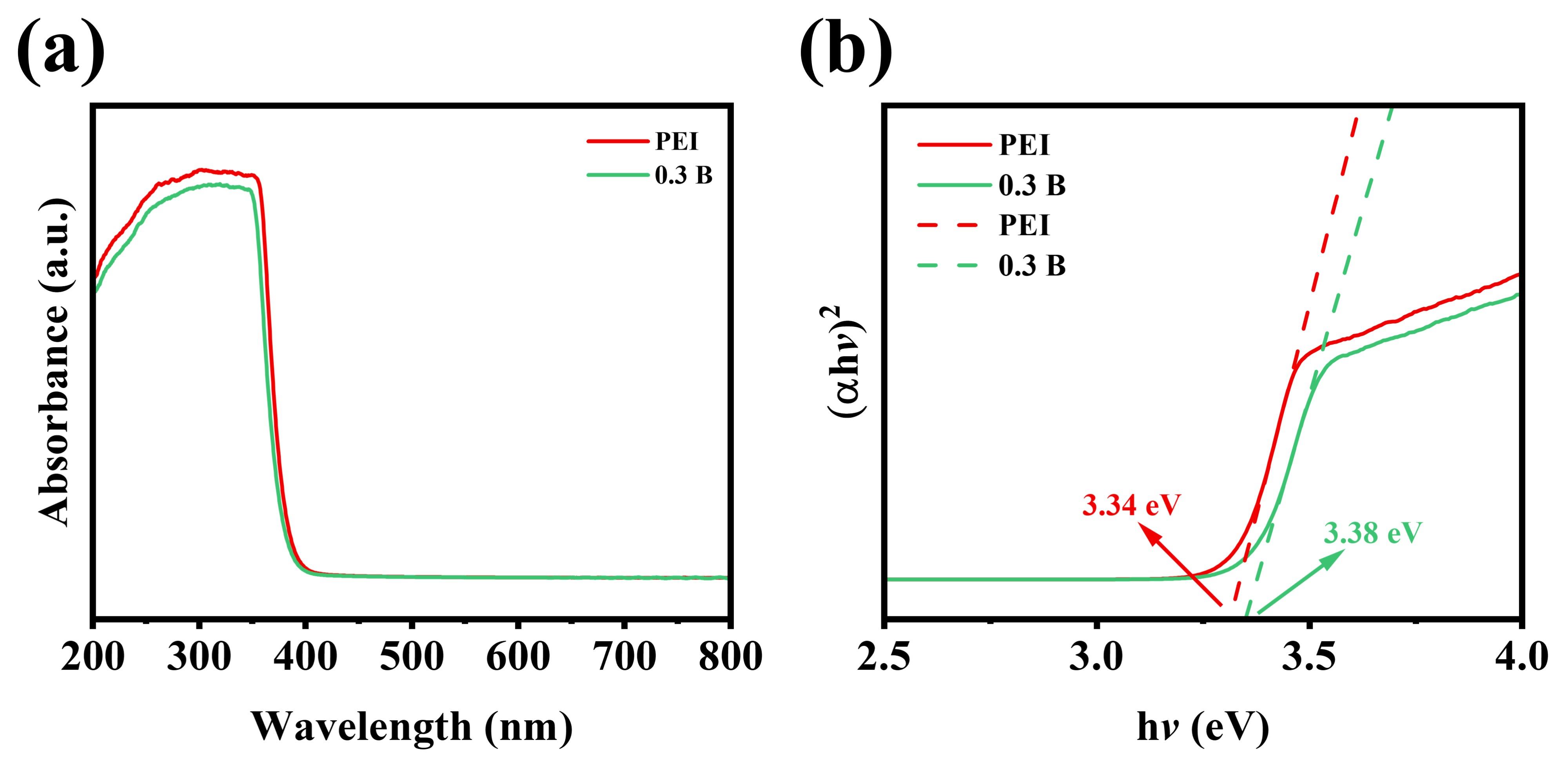


**Figure S17.** UV-vis spectra and band gap calculation curves of PEI composite films. (a) UV-vis spectra of PEI composite film; (b) Band gap calculated from the UV-vis spectra of PEI composite film.


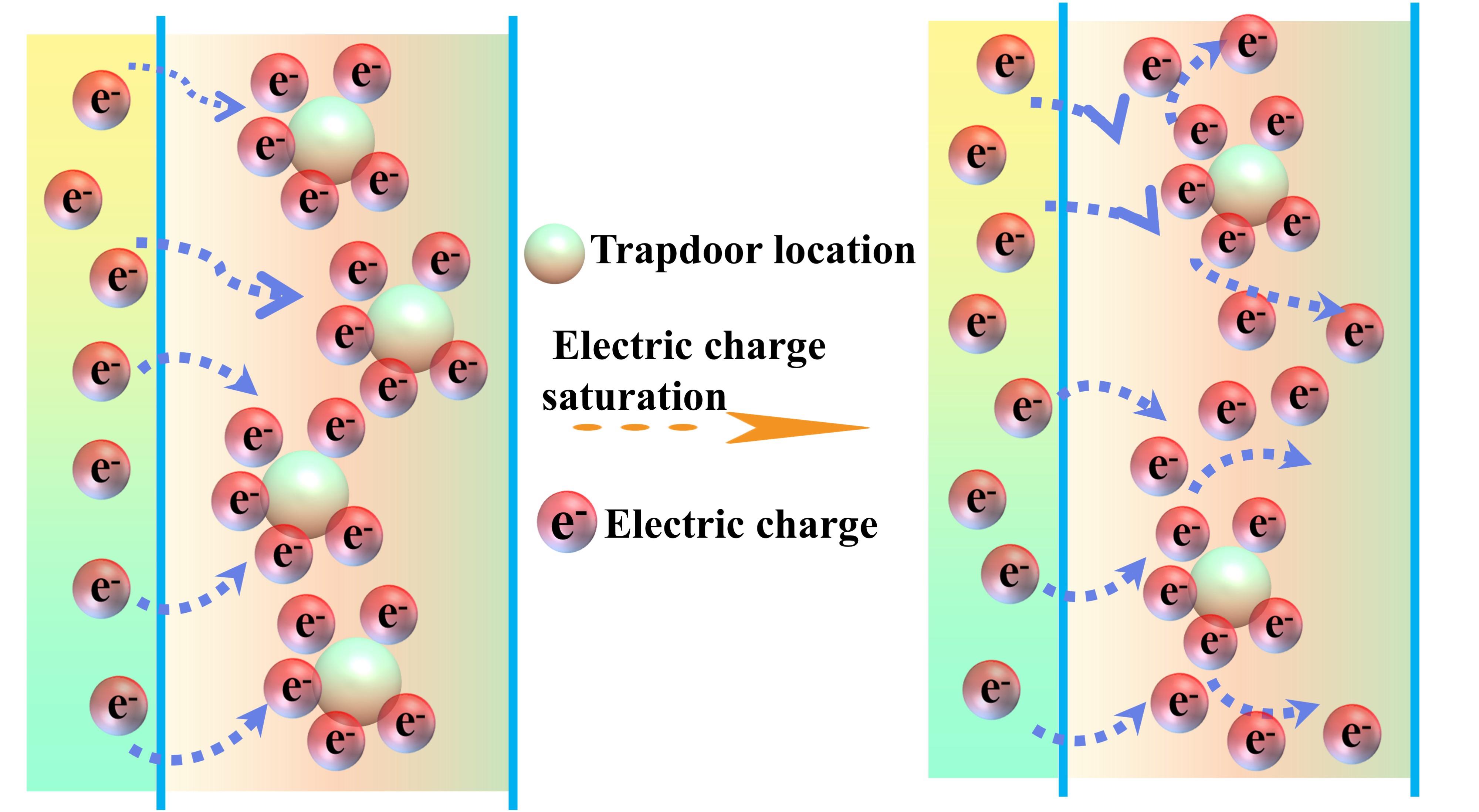


**Figure S18.** Internal modeling of charge trapping.


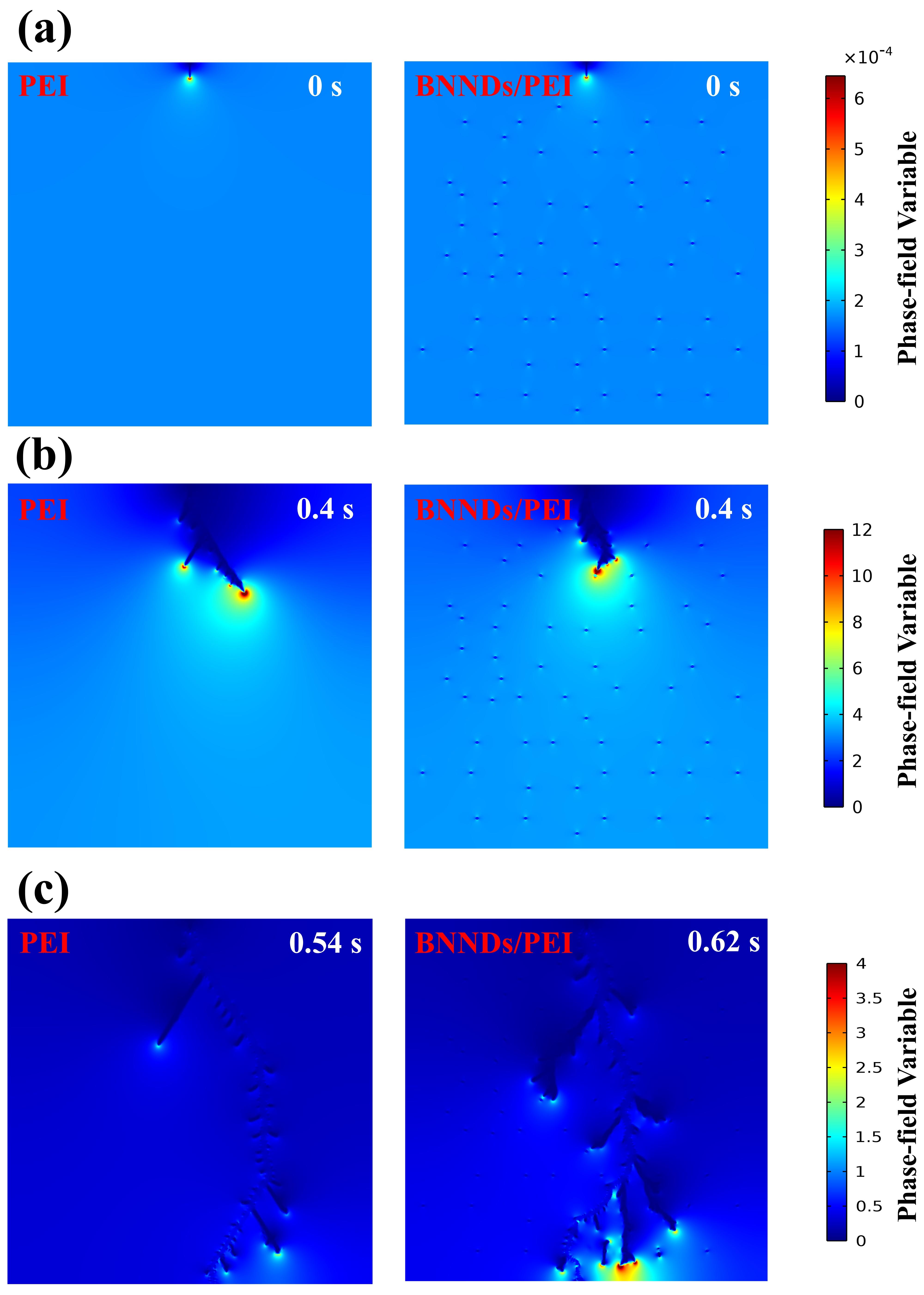


**Figure S19.** Electrical dendrite evolution of composite films at different times. Electrical tree paths in composite film and PEI film at 0.3 s (a) and 0.4 s (b); (c) Electrical tree paths in composite film and PEI film after breakdown.


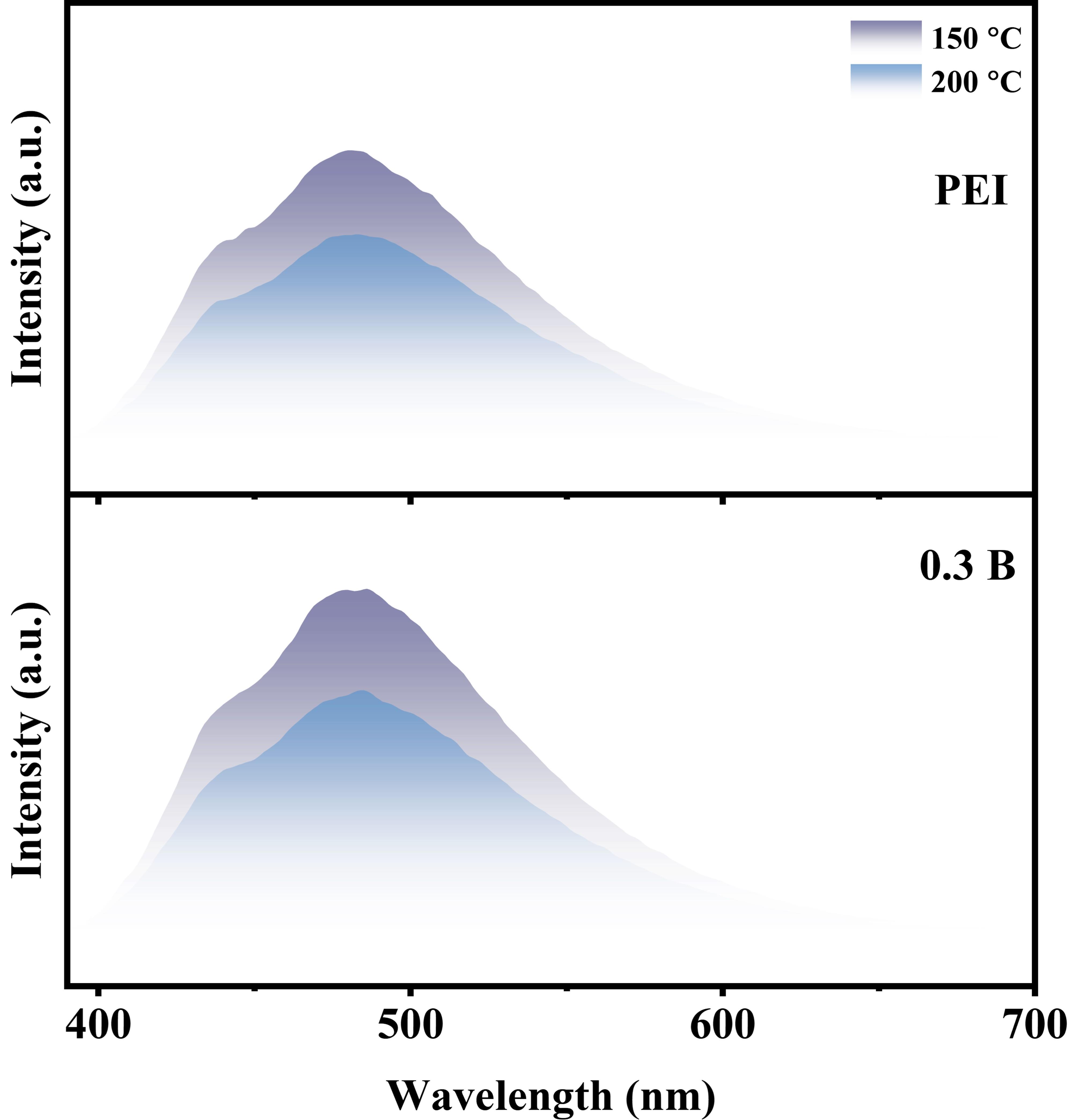


**Figure S20.** Temperature-dependent fluorescence emission spectra of PEI and 0.3 B films with an excitation wavelength of 375 nm.


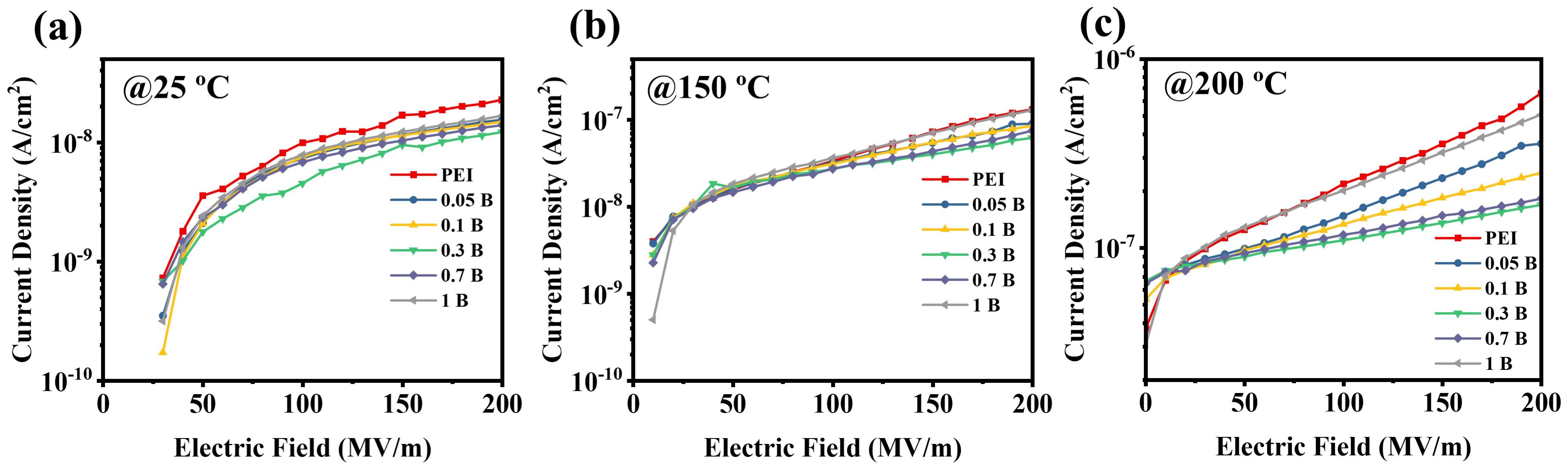


**Figure S21.** Leakage current density of PEI composite films at different temperatures. (a) 25 ºC; (b) 150 ºC; (c) 200 ºC.


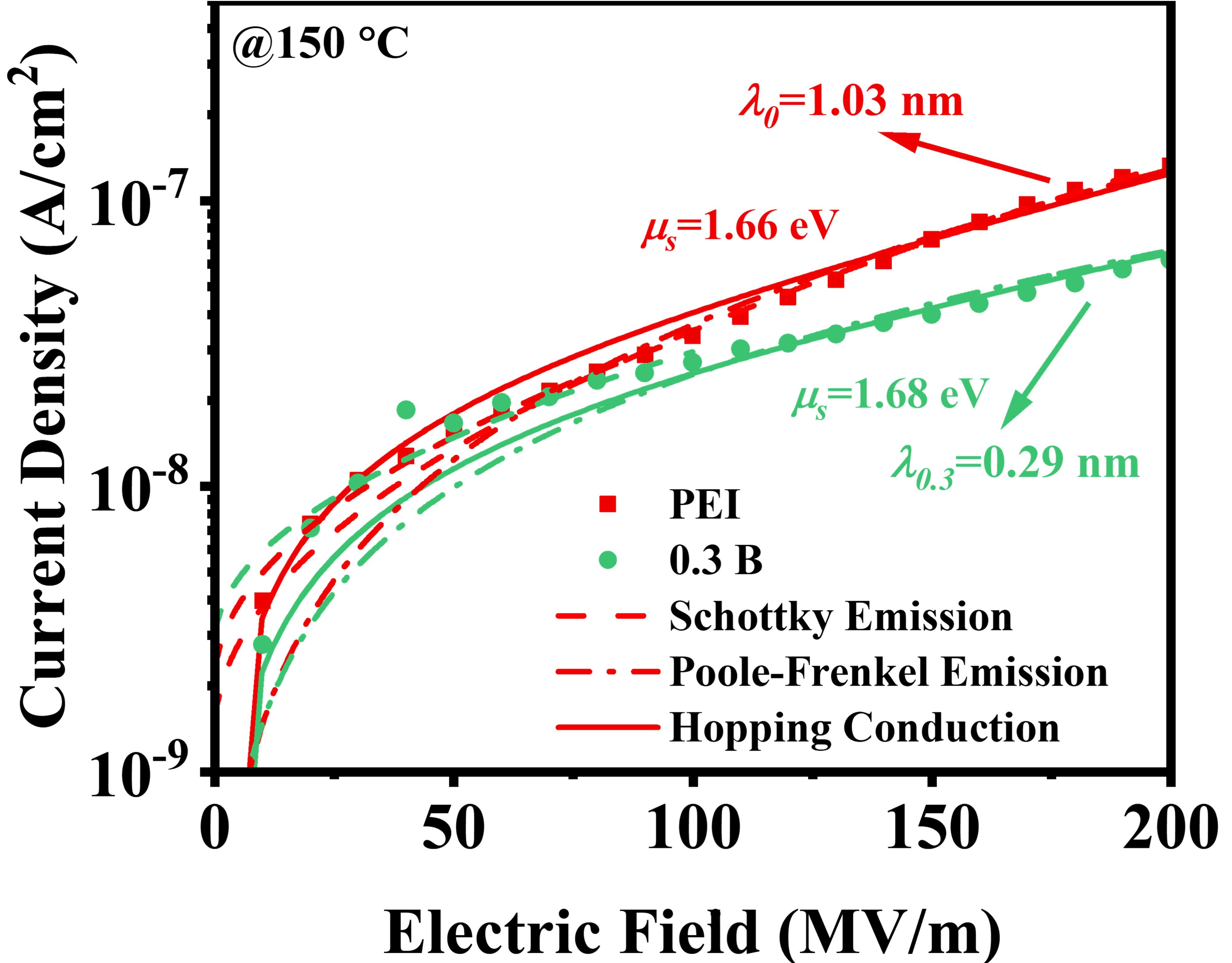


**Figure S22.** Leakage current fitting of PEI composite films at 150 ºC.


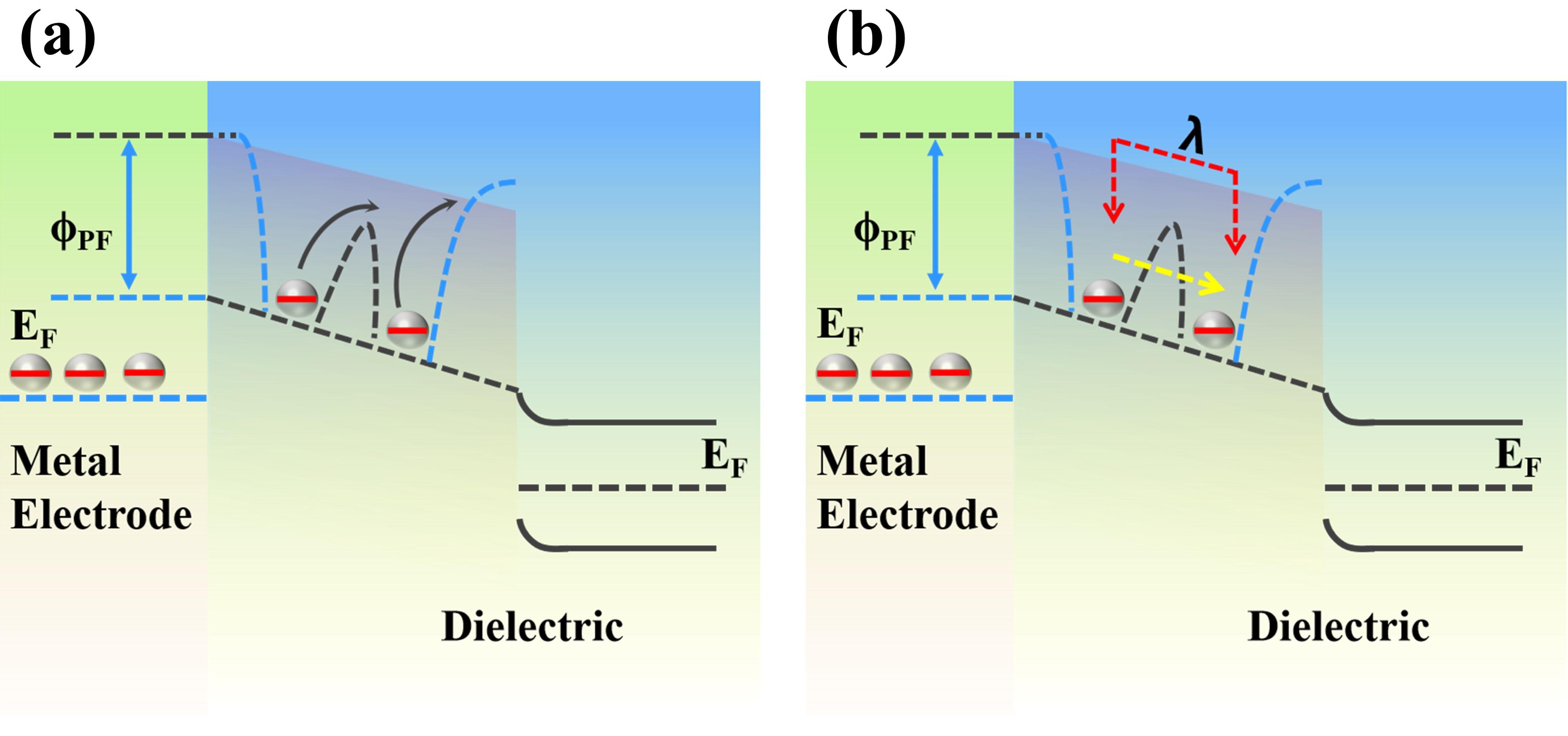


**Figure S23.** The charge transfer mechanism within the thin film. (a) Diagrams of Poole-Frenkel emission conduction mechanism at the interface of composite film-metal electrodes at high temperatures; (b) Diagrams of hopping conduction mechanism inside composite films at elevated temperatures.


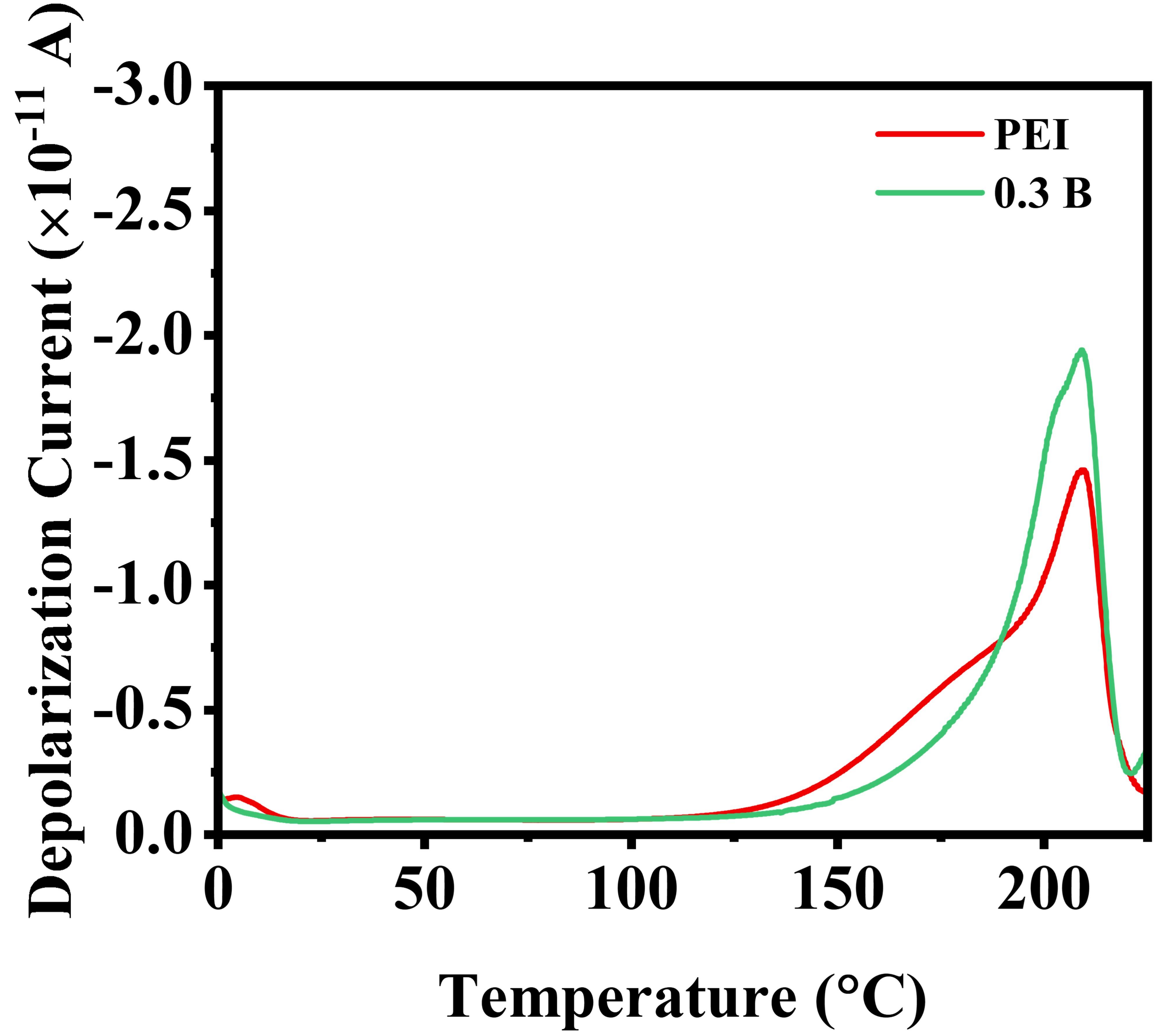


**Figure S24.** TSDC curves of PEI composite films.


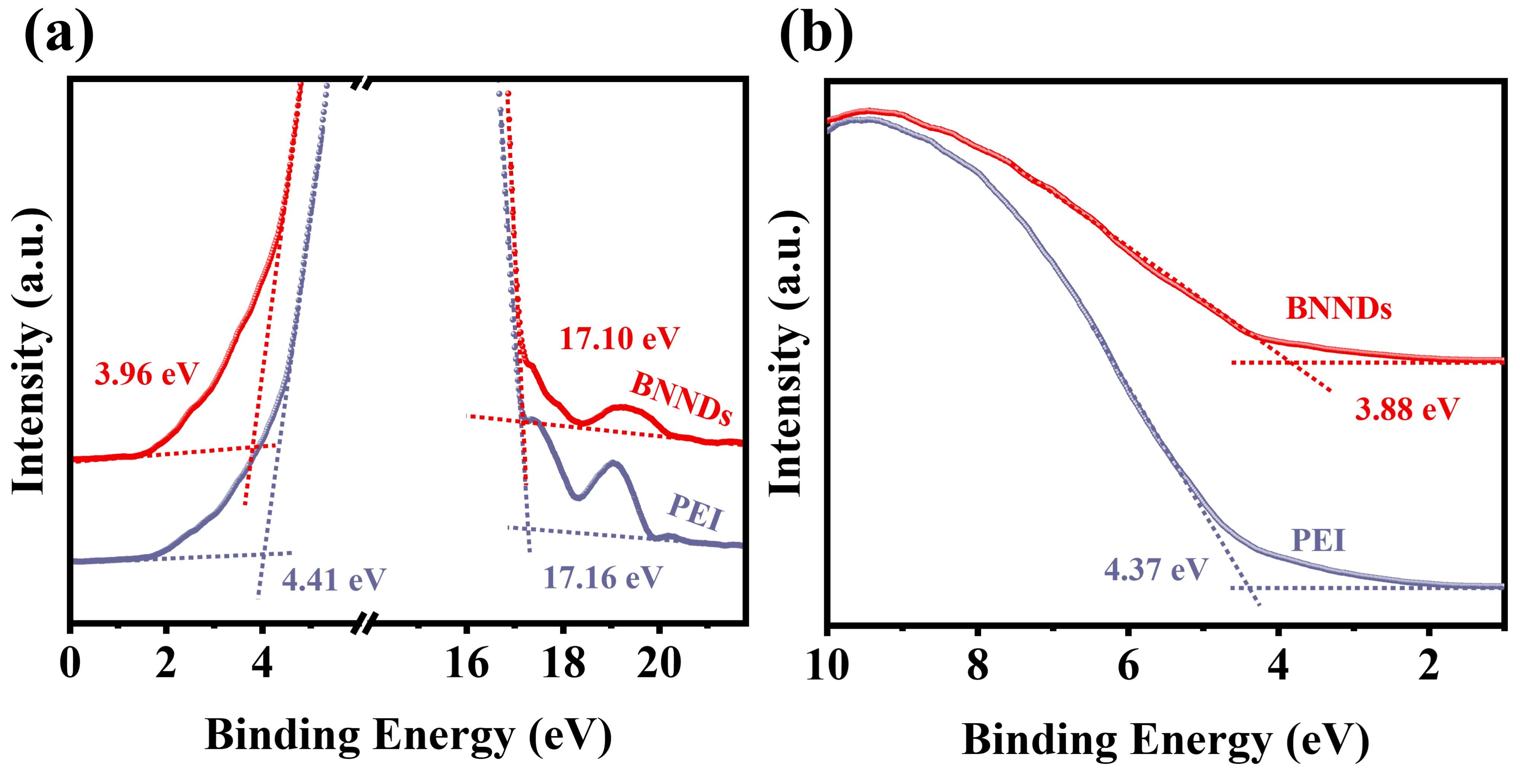


**Figure S25.** (a) UPS curves and (b) valence band analysis for PEI as well as BNNDs.


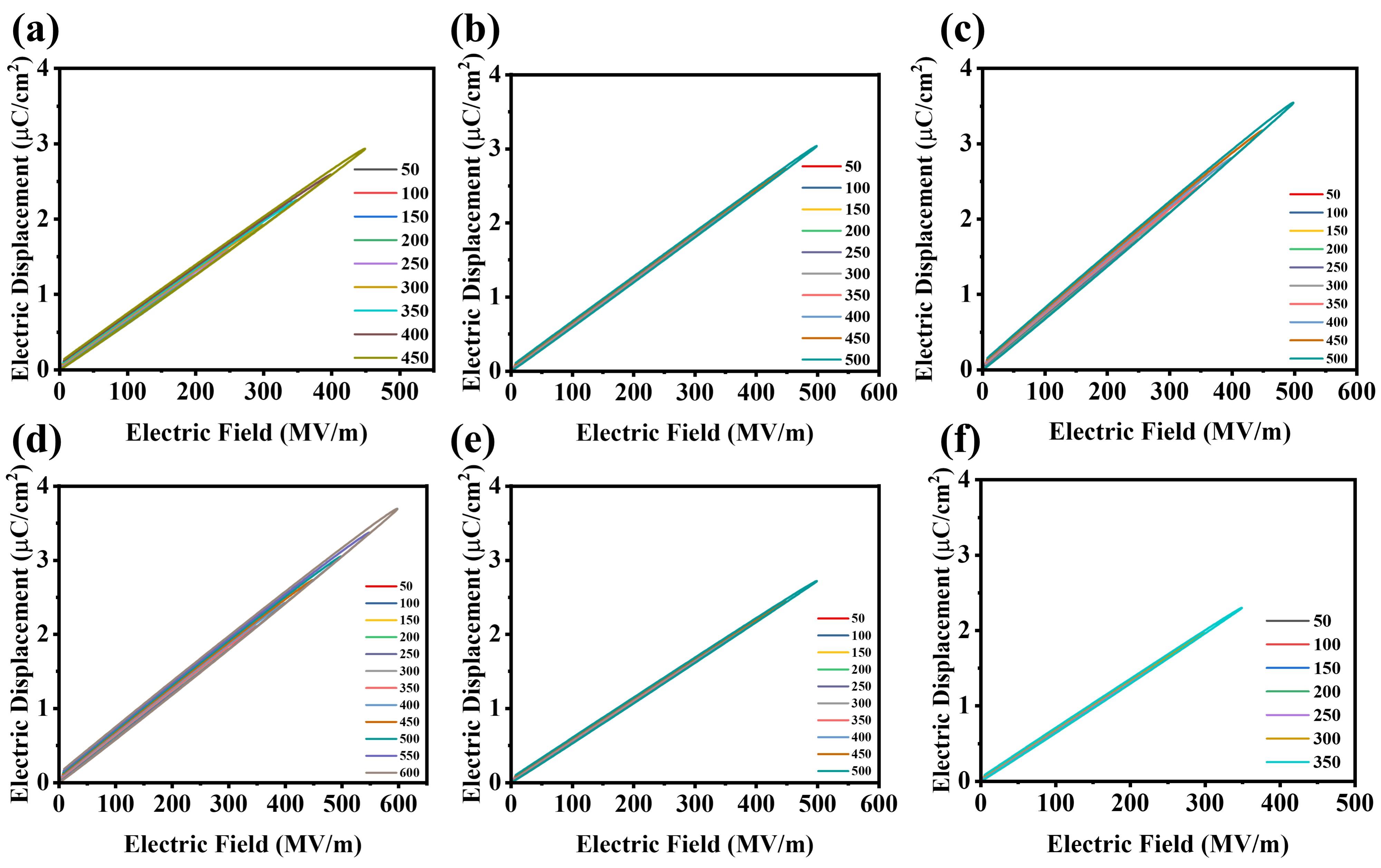


**Figure S26.** D-E curves of PEI composite films obtained by testing at room temperature. (a) PEI; (b) 0.05 B; (c) 0.1 B; (d) 0.3 B; (e) 0.7 B; (f) 1 B.


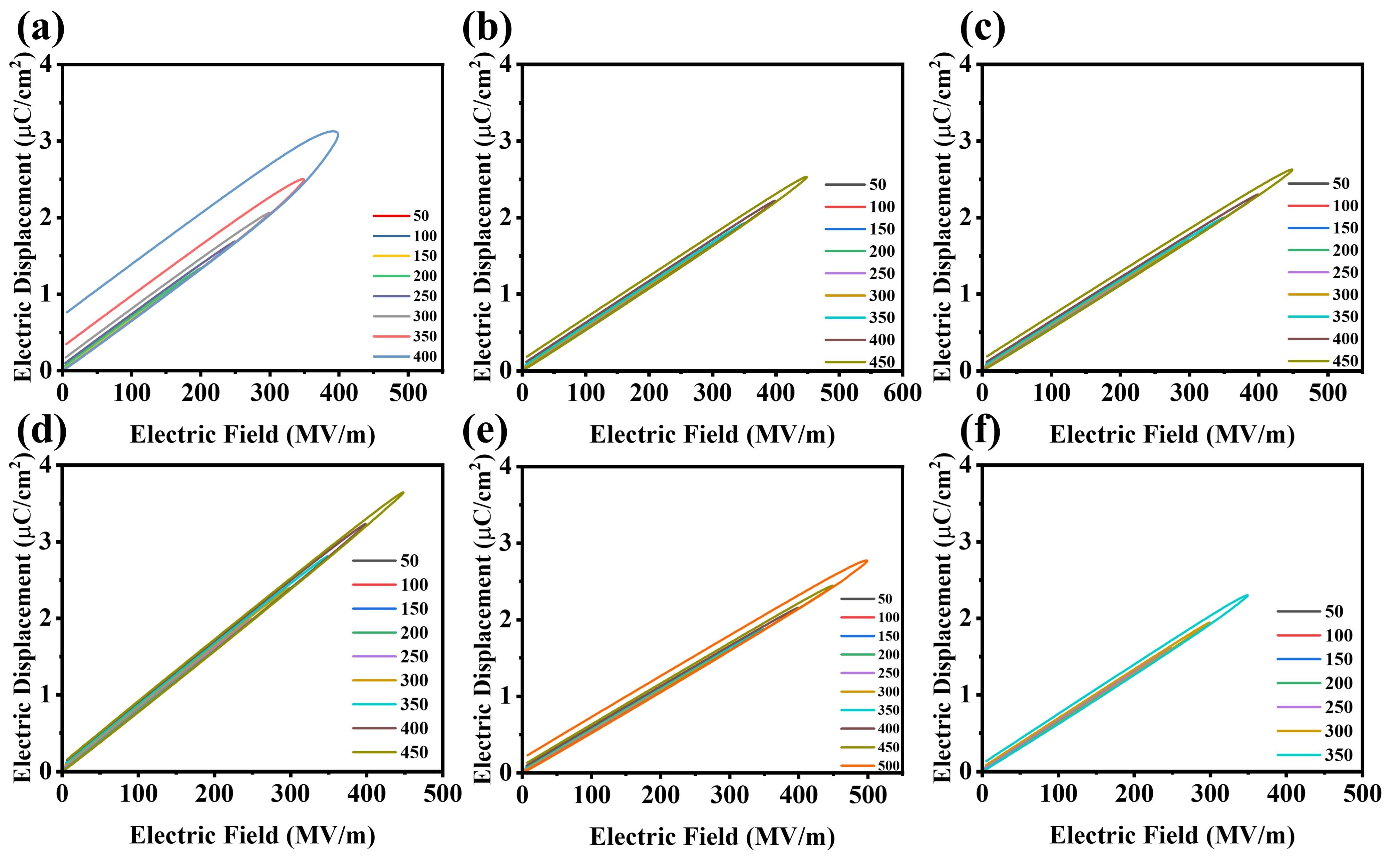


**Figure S27.** D-E curves of PEI composite films obtained by testing at 150 ºC. (a) PEI; (b) 0.05 B; (c) 0.1 B; (d) 0.3 B; (e) 0.7 B; (f) 1 B.


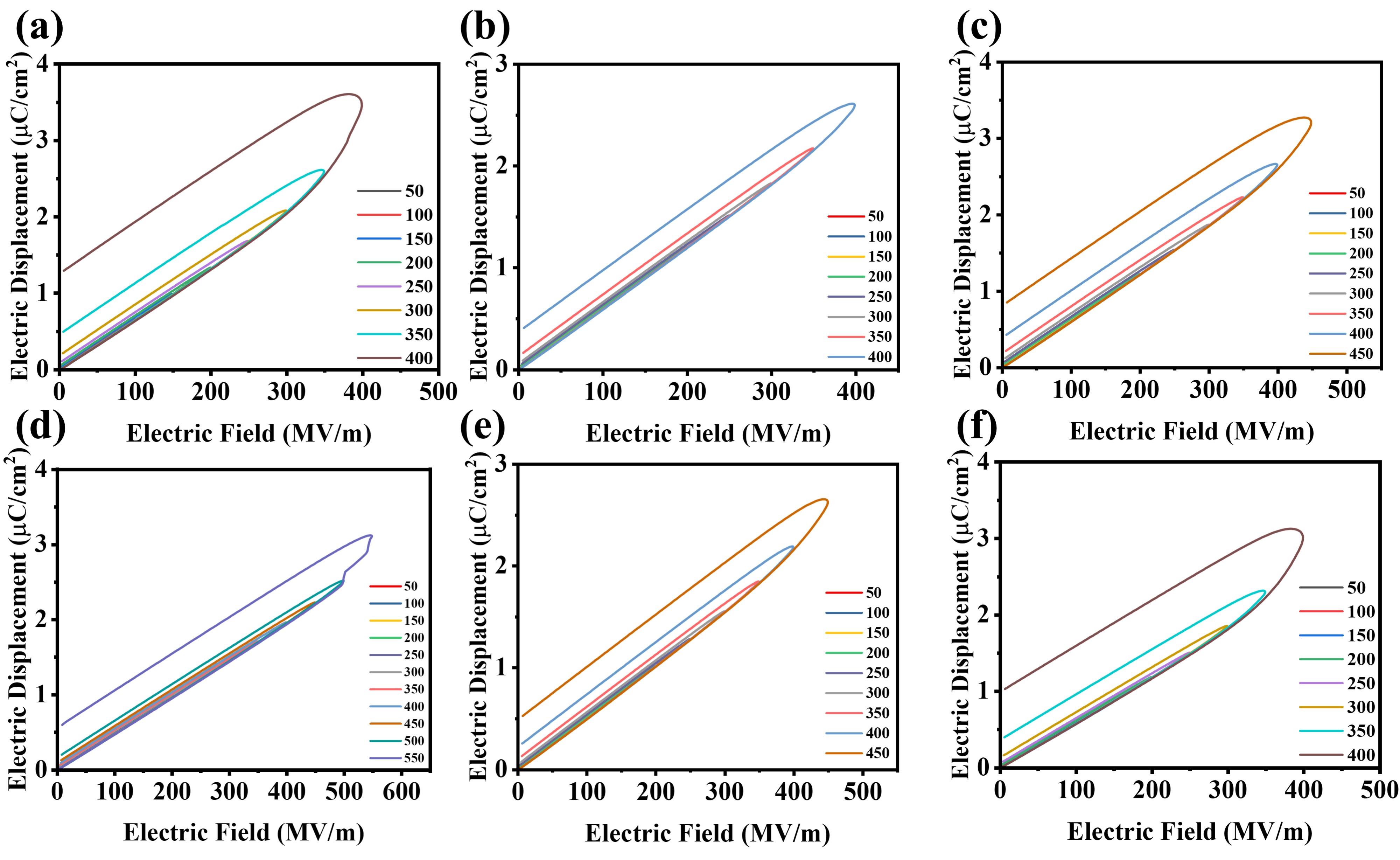


**Figure S28.** D-E curves of PEI composite films obtained by testing at 200 ºC. (a) PEI; (b) 0.05 B; (c) 0.1 B; (d) 0.3 B; (e) 0.7 B; (f) 1 B.


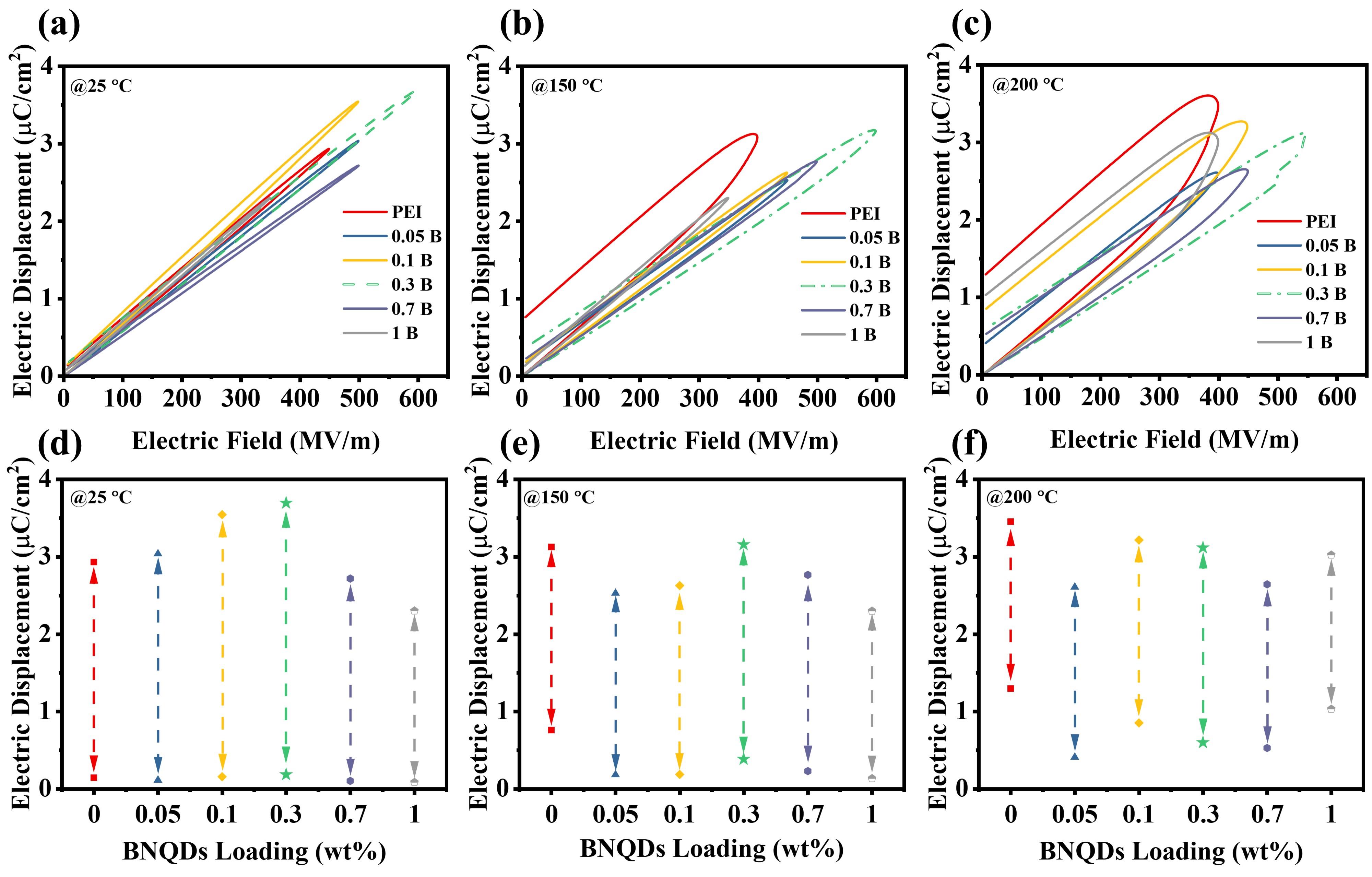


**Figure S29.** D-E curves and net polarization analysis of PEI composite films at different temperatures and maximum electric fields.


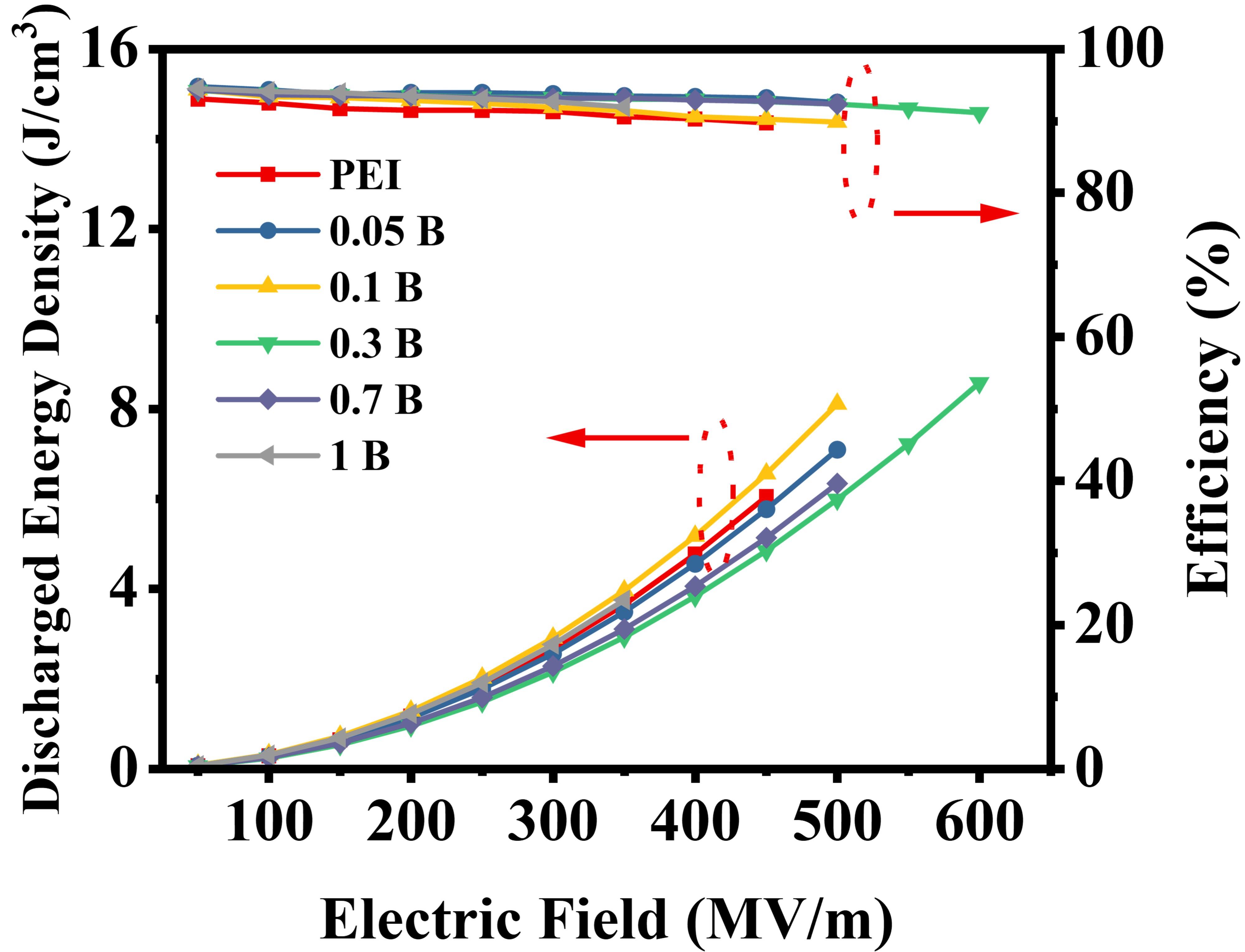


**Figure S30.** Discharge energy density and efficiency of PEI composite films at room temperature.


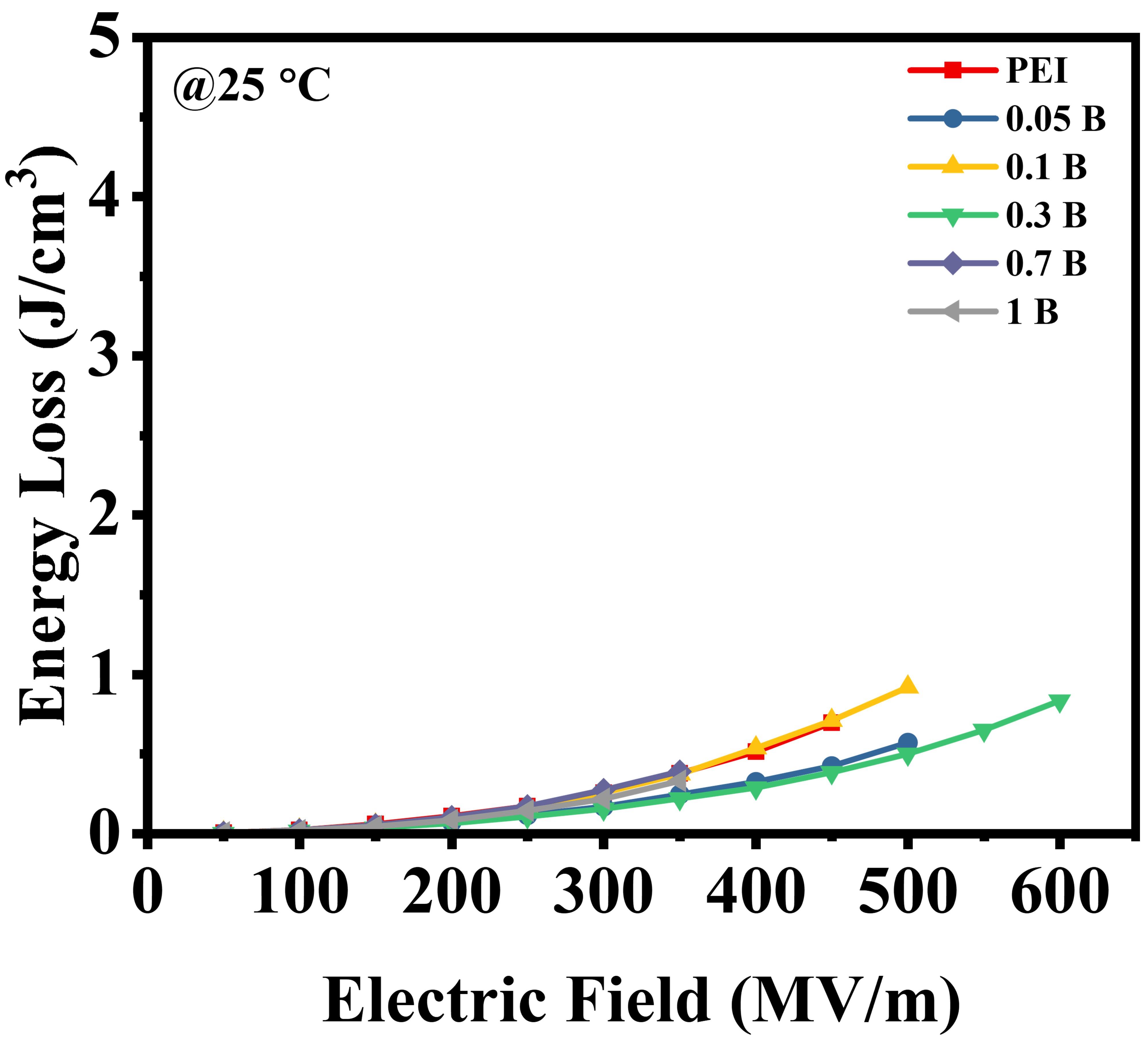


**Figure S31.** Energy loss analysis of PEI composite films at room temperature.


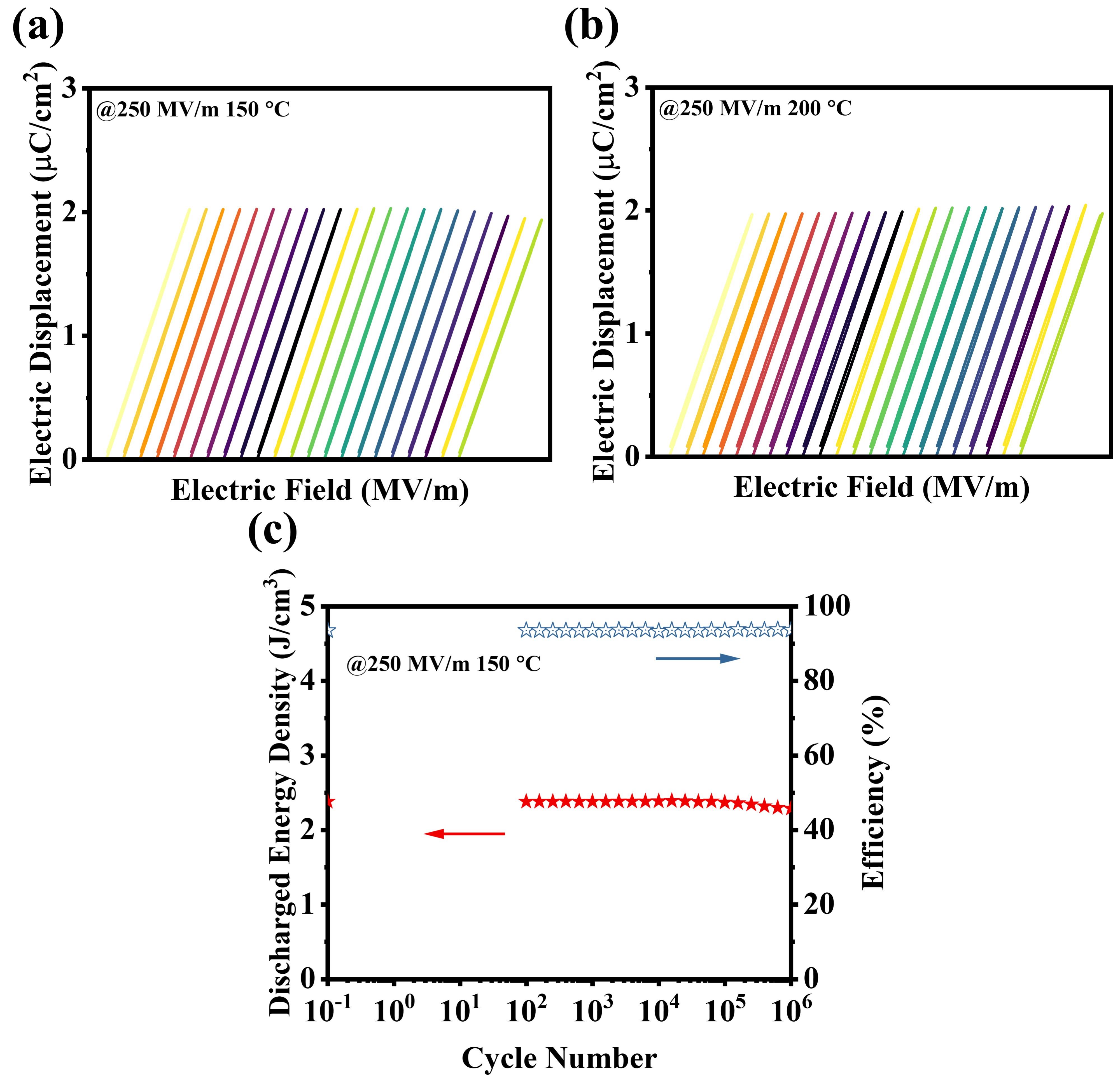


**Figure S32.** Cycling performance of 0.3 B film at different temperatures. (a) D-E curves at 150 ºC; (b) D-E curves at 200 ºC; (c) Cycling energy storage capacity and efficiency at 150 ºC.


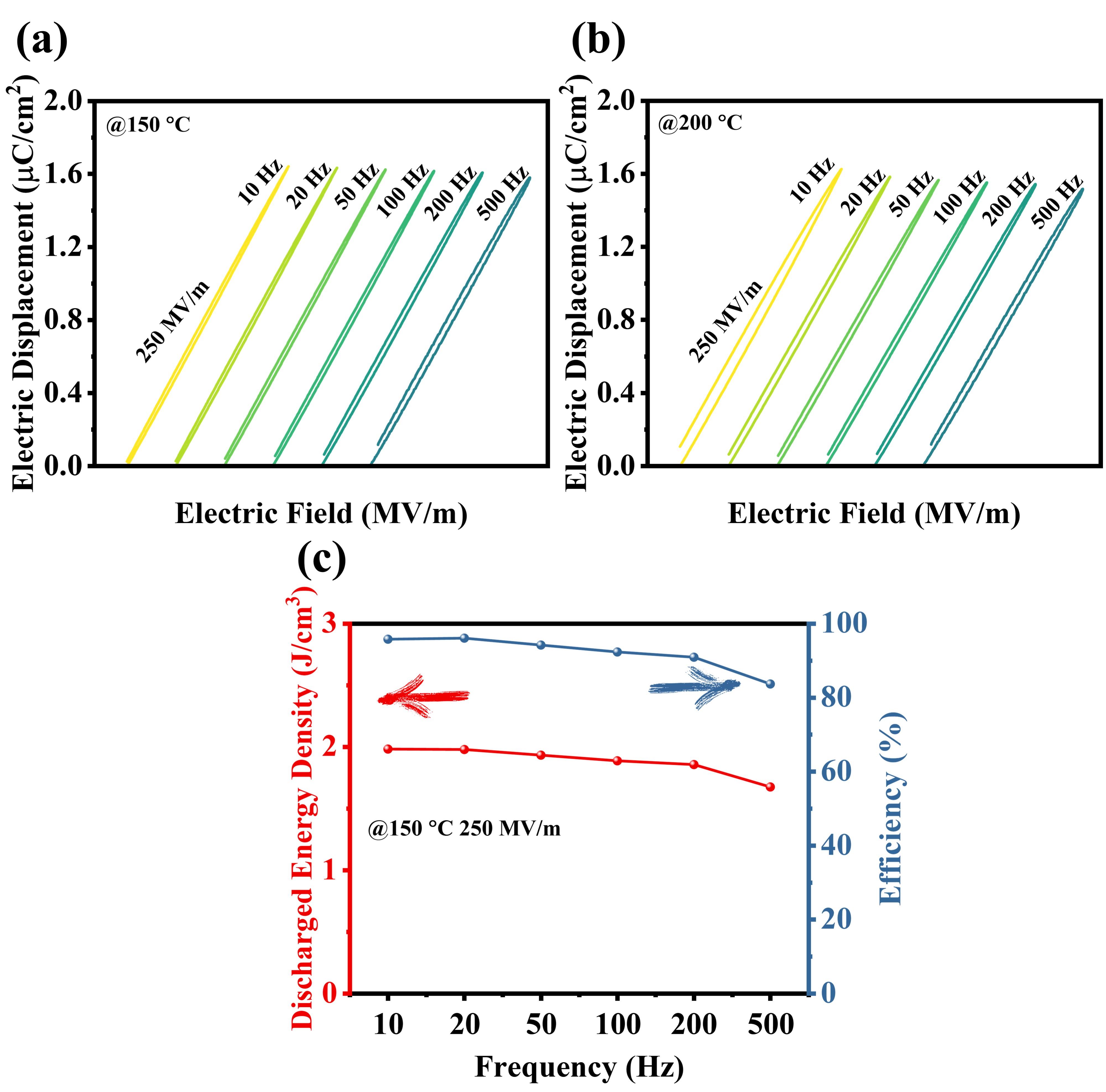


**Figure S33.** Analysis of energy storage behavior of 0.3 B film at different electric field frequencies. (a) D-E curves at different frequencies at 150 ºC; (b) D-E curves at different frequencies at 200 ºC; (c) Energy storage density and efficiency at different frequencies at 150 ºC.


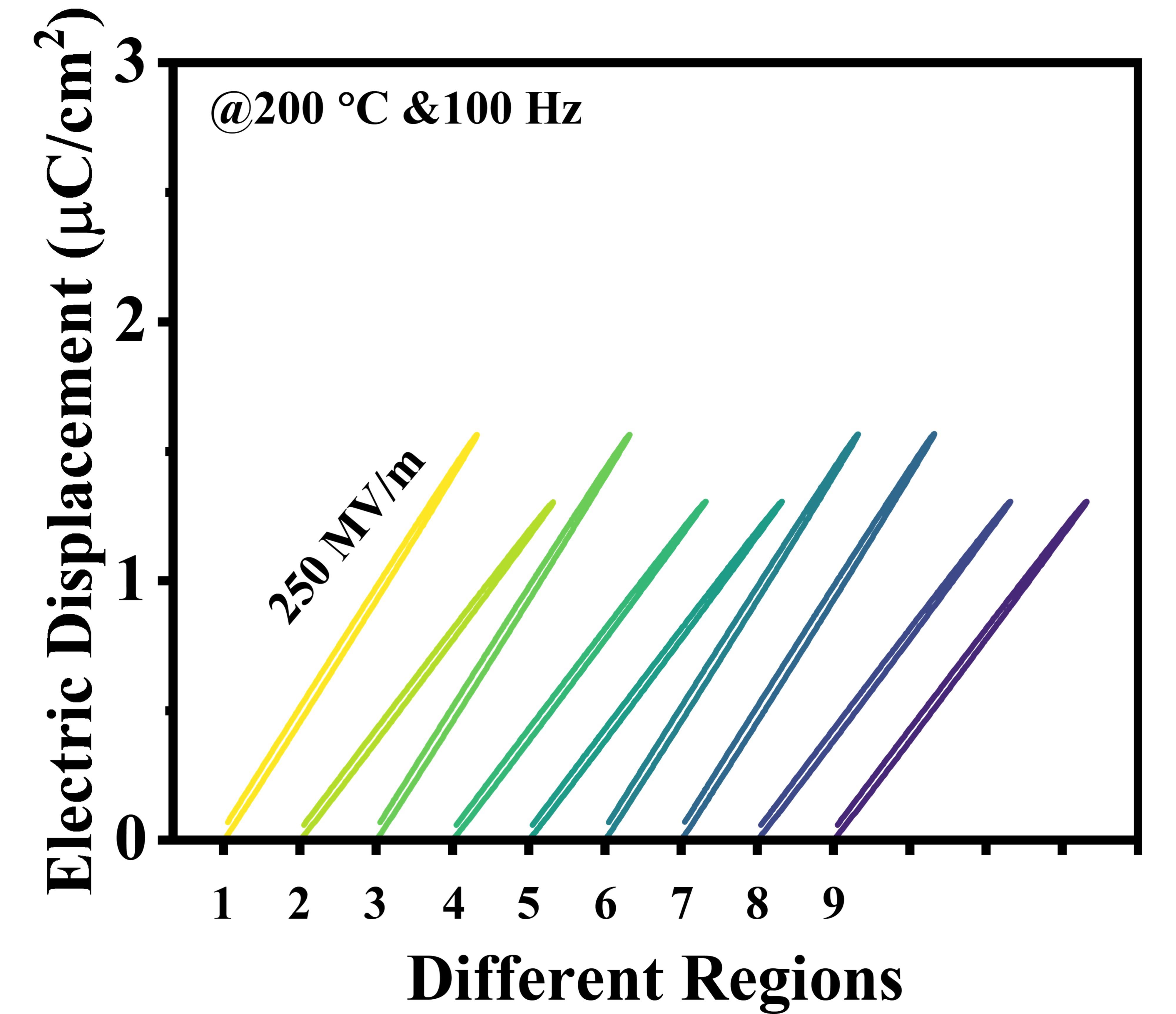


**Figure S34.** D-E curves measured at different regions of the composite film.


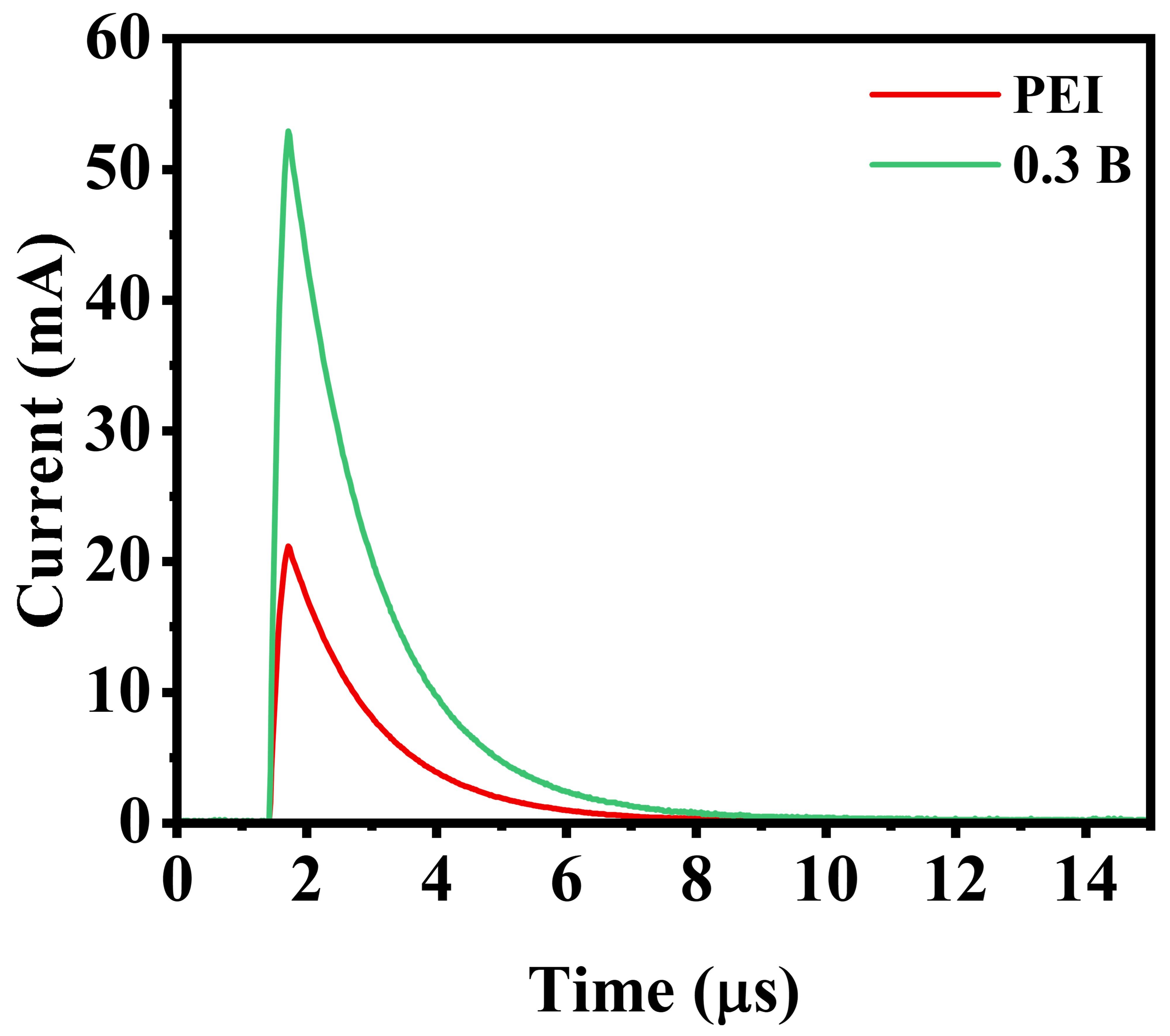


**Figure S35.** Current curves of PEI and 0.3 B film in the discharged state.


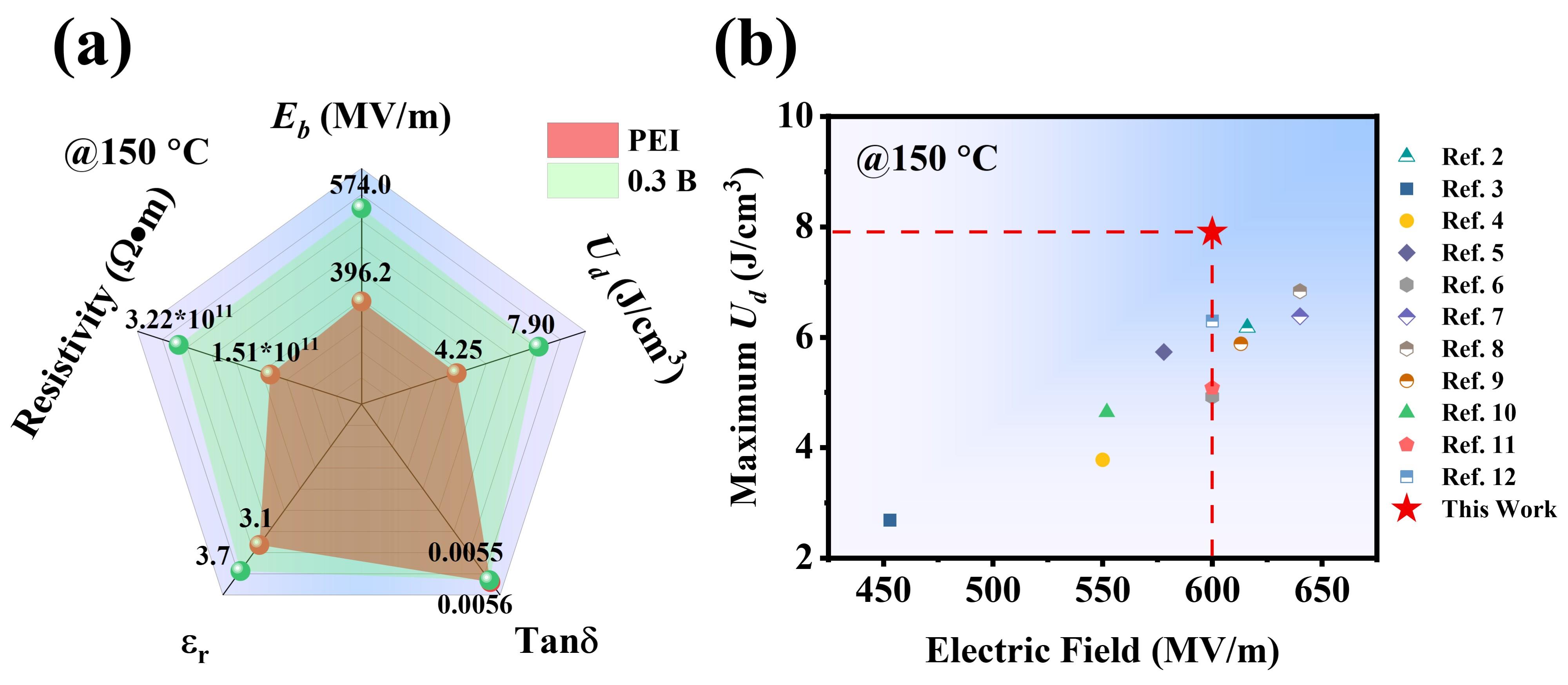


**Figure S36.** (a) Comparison of the properties of PEI and 0.3 B film; (b) Comparison with the properties reported in the current articles [2-12].

**Table S1.** TSDC results of PEI composite films.

|  | E_1_  (×10 eV) | E_2_  (×10 eV) | Q_1_  (nC) | Q_2_  (nC) | T_peak-max1_  (ºC) | T_peak-max2_  (ºC) |
| --- | --- | --- | --- | --- | --- | --- |
| 0 | 0.15 | 0.70 | 0.14 | 0.35 | 187.1 | 207.90 |
| 0.3 B | 0.20 | 0.60 | 0.23 | 0.26 | 192.14 | 206.79 |

**References**

[1] W. Zhang, D. Ai, S. Fan, R. Yang, X. Du, X. Yang, F. Lv, Y. Liu, Y. Cheng, X. Yu, Tailoring a Dual Crosslinking Network in All-Organic Aramid Composite Film for Superior High-Temperature Capacitive Energy Storage, *Energy Storage Mater.* **2025**, 77, 104180.

[2] Y. Zhou, Z. Zhang, Q. Tang, X. Ma, X. Hou, Enhancing the High-Temperature Energy Storage Properties of PEI Dielectrics by Constructing Trap-Rich Covalently Cross-Linked Networks via POSS-Functionalized BNNS, *Mater. Horiz.* **2024**, 11, 4348-4358.

[3] J. Pei, J. Zhu, L. Yin, Y. Zhao, M. Yang, S. Zhong, Q. Feng, Z. Dang, Flexible High-Temperature Polymer Dielectrics Induced by Ultraviolet Radiation for High Efficient Energy Storage, *Adv. Funct. Mater.* **2024**, 34, 2316869.

[4] X. Dong, Y. Wang, Y. Cao, N. Li, J. Fu, Y. Wang, J. Yu, Z. Hu, Enhanced High-Temperature Energy Storage Performance in All-Organic Dielectric Films Through Synergistic Crosslinking of Chemical and Physical Interaction, *Chem. Eng. J.* **2024**, 500, 157312.

[5] X. Yu, R. Yang, W. Zhang, X. Yang, C. Ma, K. Sun, G. Shen, F. Lv, S. Fan, Interface Engineering of Polymer Composite Films for High-Temperature Capacitive Energy Storage, *Chem. Eng. J.* **2024**, 496, 154056.

[6] B. Peng, P. Wang, H. Luo, G. He, H. Xie, Y. Liu, S. Chen, X. Li, Y. Wan, R. Guo, Outstanding High-Temperature Capacitive Performance in All-Organic Dielectrics Enabled by Synergistic Optimization of Molecular Traps and Aggregation Structures, *Mater. Horiz.* **2025**, 12, 1223-1233.

[7] Y. Shang, Y. Feng, Z. Meng, C. Zhang, T. Zhang, Q. Chi, Achieving Synergistic Improvement in Dielectric and Energy Storage Properties at High-Temperature of All-Organic Composites via Physical Electrostatic Effect, *Mater. Horiz.* **2024**, 11, 1528-1538.

[8] Z. Wang, Y. Zhao, M. Yang, H. Yan, C. Xu, B. Tian, C. Zhang, Q. Xie, Z. Dang, Surface Strengthening of Polymer Composite Dielectrics for Superior High-Temperature Capacitive Energy Storage, *Adv. Energy Mater.* **2025**, 15, 2405411.

[9] N. Zhang, H. Zhao, C. Zhang, H. Guo, Z. Dang, J. Bai, Ultrafine MOF as Charge Trap Enables Superior High-Temperature Energy Storage Performance in Polyetherimide Composites Dielectrics, *Chem. Eng. J.* **2025**, 508, 161063.

[10] C. Yuan, Y. Zhou, Y. Zhu, J. Liang, S. Wang, S. Peng, Y. Li, S. Cheng, M. Yang, J. Hu, B. Zhang, R. Zeng, J. He, Q. Li, Polymer/Molecular Semiconductor All-Organic Composites for High-Temperature Dielectric Energy Storage, *Nat. Commun.* **2020**, 11, 3919.

[11] B. Zhang, X. Chen, Z. Pan, P. Liu, M. Mao, K. Song, Z. Mao, R. Sun, D. Wang, S. Zhang, Superior High-Temperature Energy Density in Molecular Semiconductor/Polymer All-Organic Composites, *Adv. Funct. Mater.* **2022**, 33, 2210050.

[12] S. Hou, Y. Liu, Y. Hao, W. Zhu, Y. Xu, J. Gao, X. Lou, L. Zhong, Side Group Induced Steric Hindrance Effect in Polyetherimide Derived Polymers for High-Temperature Capacitive Energy Storage Performance, *Chem. Eng. J.* **2025**, 519, 165324.

1. * Corresponding author.

   E-mail: yangjinghui@swjtu.edu.cn (J.H. Yang), yongwang1976@swjtu.edu.cn (Y. Wang) [↑](#footnote-ref-0)
